# Revisão Sistemática Sobre a Eficácia do Atenolol no Tratamento Anti-Hipertensivo: Recomendação da Sociedade Brasileira de Cardiologia

**DOI:** 10.36660/abc.20250034

**Published:** 2025-09-08

**Authors:** Andréa Araujo Brandão, Cibele Isaac Saad Rodrigues, Wilson Nadruz, Paulo Cesar B Veiga Jardim, Fernando Nobre, Sergio Emanuel Kaiser, Otavio Rizzi Coelho, Fernanda Marciano Consolim Colombo, Leonardo Castro Luna, Andressa Braga, Quenia Dias Morais, Luiz Bortolotto

**Affiliations:** 1 Universidade do Estado do Rio de Janeiro Rio de Janeiro RJ Brasil Universidade do Estado do Rio de Janeiro, Rio de Janeiro, RJ – Brasil; 2 Pontifícia Universidade Católica de São Paulo Sorocaba SP Brasil Pontifícia Universidade Católica de São Paulo, Sorocaba, SP – Brasil; 3 Universidade Estadual de Campinas Campinas SP Brasil Universidade Estadual de Campinas, Campinas, SP – Brasil; 4 Universidade Federal de Goiás Goiânia GO Brasil Universidade Federal de Goiás, Goiânia, GO – Brasil; 5 Universidade de São Paulo Ribeirão Preto SP Brasil Universidade de São Paulo (USP), Ribeirão Preto, SP – Brasil; 6 Universidade do Estado do Rio de Janeiro Clinex Rio de Janeiro RJ Brasil Universidade do Estado do Rio de Janeiro – Clinex, Rio de Janeiro, RJ – Brasil; 7 Universidade Estadual de Campinas Campinas SP Brasil Universidade Estadual de Campinas (UNICAMP), Campinas, SP – Brasil; 8 Hospital das Clínicas Faculdade de Medicina Universidade de São Paulo São Paulo SP Brasil Instituto do Coração do Hospital das Clínicas da Faculdade de Medicina da Universidade de São Paulo, São Paulo, SP – Brasil; 9 Instituto Nacional de Cardiologia Rio de Janeiro RJ Brasil Instituto Nacional de Cardiologia, Rio de Janeiro, RJ – Brasil

**Keywords:** Hipertensão, Revisão Sistemática, Metanálise, Tratamento Farmacológico, Atenol

## Abstract

**Fundamento:**

A hipertensão arterial (HA) é um problema global de saúde pública, com alta prevalência e impacto significativo na morbimortalidade cardiovascular. Betabloqueadores cardiosseletivos, como o atenolol, são amplamente utilizados no tratamento da HA, mas sua indicação como terapia de primeira linha é controversa.

**Objetivos:**

Avaliar a eficácia e a segurança do atenolol no tratamento da HA primária, em comparação com outras classes de medicamentos anti-hipertensivos de primeira escolha.

**Métodos:**

Foi realizada uma revisão sistemática com base em uma pergunta estruturada no formato PICO. Foram incluídos ensaios clínicos randomizados que compararam o atenolol com outros anti-hipertensivos. A busca foi conduzida em três bases de dados internacionais. A qualidade metodológica foi avaliada por meio da ferramenta RoB 2, e a certeza da evidência, pelo sistema GRADE. O desfecho primário composto foi a ocorrência de eventos cardiovasculares maiores. Desfechos secundários incluíram mortalidade por todas as causas, infarto do miocárdio e acidente vascular cerebral, analisados separadamente.

**Resultados:**

Sete ensaios clínicos atenderam aos critérios de inclusão. Em comparação com anlodipino e losartana, o atenolol apresentou incidência discretamente maior de eventos cardiovasculares, com certeza da evidência baixa e moderada, respectivamente. A combinação de hidroclorotiazida e amilorida demonstrou maior redução de eventos cardiovasculares em relação ao atenolol, embora com certeza da evidência muito baixa. A redução da pressão arterial (PA) foi semelhante entre os medicamentos comparados.

**Conclusões:**

Apesar das limitações das evidências disponíveis, o atenolol demonstrou eficácia semelhante na redução da PA, com pequenas diferenças em desfechos cardiovasculares, favorecendo outras classes de anti-hipertensivos. Sua prescrição pode ser considerada entre as opções de associação medicamentosa no tratamento da HA primária em adultos. Outros betabloqueadores não foram avaliados nesta revisão sistemática.

## Graphical abstract

**Figura Central:**
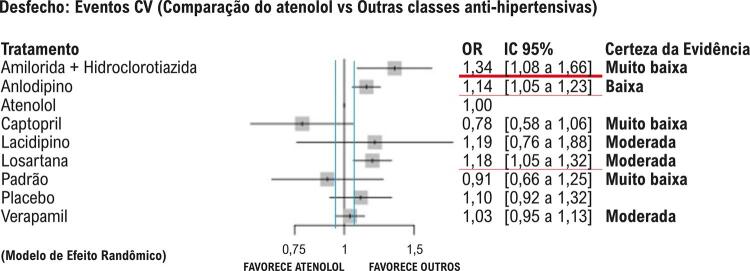
Revisão Sistemática Sobre a Eficácia do Atenolol no Tratamento Anti-Hipertensivo: Recomendação da Sociedade Brasileira de Cardiologia

## Introdução

A hipertensão arterial (HA) é um dos principais problemas de saúde pública global e nacional, sendo responsável por aproximadamente 10 milhões de mortes por ano no mundo, principalmente devido às complicações cardiovasculares, como infarto agudo do miocárdio (IAM) e acidente vascular cerebral (AVC).^1^ No Brasil, as prevalências de HA observadas foram: 21,4% (IC 95%: 20,8 a 22,0) com base no critério de autorrelato; 22,8% (IC 95%: 22,1 a 23,4) para pressão arterial (PA) aferida; e 32,3% (IC 95%: 31,7 a 33,0) quando considerada a PA aferida e/ou o relato de uso de medicamento(s) anti-hipertensivos.^2^ Estratégias efetivas de prevenção, diagnóstico precoce e controle da HA são essenciais para mitigar seu impacto, especialmente em populações vulneráveis.^3^

O tratamento medicamentoso da HA ainda hoje é um grande desafio. Estudos proeminentes sobre o tema, como o ACCORD^4^ e o SPRINT,^5^ que estabeleceram metas de controle pressórico mais intensivas nos grupos de intervenção (PA sistólica <120 mmHg), demonstraram que, em média, o indivíduo com HA precisará de aproximadamente três medicamentos associados para atingir esse objetivo. Além de impactar nos custos e na adesão dos pacientes ao tratamento medicamentoso, esse fato também reforça a necessidade de um número maior de opções terapêuticas com eficácia clínica comprovada.

Betabloqueadores (BBs) são um grupo heterogêneo de medicamentos que atuam por meio do bloqueio dos receptores beta-adrenérgicos periféricos e centrais, além de inibirem a liberação de renina pelo aparelho justa-glomerular renal, mas diferem quanto à seletividade e aos efeitos adicionais.^6,7^ Introduzidos para uso clínico na década de 1960, constituíam a classe mais prescrita para HA nos anos 1980.^8,9^ Posteriormente, foram publicados, nos anos 2000, estudos primários e metanálises sugerindo uma menor eficácia desses fármacos quando comparados, de forma agrupada, a outras classes anti-hipertensivas, especialmente na proteção cerebrovascular, relegando os BB a uma posição secundária no tratamento da HA.^10,11^ O questionamento atual é que esses trabalhos partiam da premissa de que os efeitos clínicos dos diferentes BB na HA seriam equivalentes e, na época, essas revisões não levavam em consideração a qualidade dos estudos (isto é, a certeza da evidência) para a elaboração de suas conclusões.

Com o objetivo de esclarecer essa lacuna no tratamento da HA, considerando as melhores evidências científicas atuais, a Sociedade Brasileira de Cardiologia (SBC) demandou o desenvolvimento de uma Recomendação Clínica sobre o uso do atenolol (o medicamento mais utilizado e disponível da classe dos BB) na população com HA.

## Métodos

Para fundamentar a Recomendação Clínica da SBC, foi realizada uma revisão sistemática. A pergunta de pesquisa, estruturada no formato PICO, foi: qual é a eficácia e segurança do tratamento da HA com atenolol em comparação a outros anti-hipertensivos? O protocolo do estudo foi registrado no International Prospective Register of Systematic Reviews (PROSPERO) sob o número CRD42024563608.

A revisão sistemática rápida, metodologia empregada neste documento, pertence à família das revisões sistemáticas, mas difere das tradicionais. Essa metodologia foi desenvolvida com o objetivo de manter um rigor metodológico adequado na busca pelas melhores evidências disponíveis, com adaptações que permitem reduzir o tempo de execução. As principais diferenças incluem: a restrição do idioma dos artigos primários ao inglês; a busca de artigos na literatura cinzenta apenas nas referências dos estudos recuperados e por meio de especialistas da área; e o uso de inteligência artificial como facilitadora da seleção e extração dos estudos. Geralmente, essas revisões são úteis para apoiar sociedades médicas ou instituições de saúde na tomada de decisões de forma sensível, transparente, sistemática e válida, com base em uma pergunta no formato PICO. Instituições de destaque na área de metodologia descreveram os métodos para esse tipo de revisão sistemática.^12-14^

A escolha do atenolol para esta revisão sistemática, em detrimento dos demais BB, foi consensual entre os autores e baseada em critérios objetivos, tais como: ser amplamente conhecido e disponível em território nacional; ser distribuído gratuitamente no componente básico do Sistema Único de Saúde; e, consequentemente, integrar a Relação Nacional de Medicamentos Essenciais (ReNaMe 2024) e o Programa Farmácia Popular do Brasil, vinculado ao Ministério da Saúde.^15,16^ Adicionalmente, é o medicamento da classe dos BB com o maior número de estudos em HA, apresentando diferença significativa em relação a outros, como metoprolol ou propranolol, que contam com poucos estudos comparativos em pacientes com HA.^11^ O efeito farmacológico do bloqueio simpático varia entre as diferentes moléculas desse grupo,^9^ e pode não ser homogêneo em relação à redução da PA e aos desfechos clínicos, razão pela qual optou-se por não agrupá-los em uma mesma análise.

Os critérios de inclusão foram: (1) revisão sistemática de ensaios clínicos randomizados (ECRs), ou ECRs originais, com pelo menos dois braços comparativos; (2) presença de atenolol como medicamento principal em um dos grupos de intervenção; (3) apresentação de dados de pelo menos um dos desfechos de interesse; (4) inclusão de pacientes adultos com 18 anos ou mais; (5) tempo de seguimento mínimo de 1 ano.

Os critérios de exclusão aplicados foram: (1) ECRs em que o atenolol fosse uma medicação de segunda escolha; (2) outros delineamentos de estudos clínicos; (3) ECRs com crossover pré-determinado; (4) estudos que fossem análises pós-hoc de ECR, sem apresentação de dados novos ou adicionais relevantes para os desfechos analisados.

O desfecho primário utilizado foi a combinação de eventos cardiovasculares maiores, incluindo morte por qualquer causa, AVC e IAM. Os desfechos secundários escolhidos foram os eventos individualizados do desfecho primário, além de eventos adversos possivelmente coletados nos estudos originais.

As buscas por estudos relacionados à pergunta PICO foram realizadas em três bases de dados: PubMed, Embase e Cochrane Library.

A seleção, extração das principais características e avaliação da qualidade foram conduzidas por dois pesquisadores experientes, de forma independente, assim como a avaliação do risco de viés, realizada por meio da ferramenta Risk of Bias 2 (RoB 2).^17^ Em caso de discordância, um terceiro metodologista era consultado. A certeza da evidência e a força das recomendações foram determinadas com base na estrutura Grading of Recommendations, Assessment, Development and Evaluations (GRADE).^18^

O sumário de efeito das comparações entre atenolol e demais medicamentos foi determinado por meio de uma metanálise em rede.

Uma metanálise em rede é uma técnica estatística avançada que combina evidências diretas e indiretas de múltiplos estudos comparativos para avaliar a eficácia de diversas intervenções simultaneamente, mesmo que nem todas tenham sido diretamente comparadas entre si.^19,20^ No entanto, esta revisão incluiu apenas comparações diretas entre atenolol e os seis tratamentos alternativos, além do braço sem tratamento. Não foi realizada nenhuma análise indireta (por exemplo, comparação entre os medicamentos A e C, a partir de estudos que compararam A com B e B com C) nem comparações em rede. A metanálise foi conduzida com o auxílio do software MetaInsight.^21^

Uma descrição detalhada da metodologia empregada nesta revisão sistemática rápida está disponível no material suplementar, assim como os Preferred Reporting Items for Systematic Reviews and Meta-Analyses (PRISMA).

Um limiar de 5% foi adotado como a diferença mínima importante (DMI) para considerar uma intervenção clinicamente relevante. Assim, se um tratamento apresentar um efeito cujo intervalo de confiança não ultrapasse a linha de neutralidade, ele será considerado estatisticamente significativo. No entanto, para que a estratégia seja considerada clinicamente relevante, o IC 95% não deve tocar a linha da DMI (5%).^22,23^ Esse limiar foi definido previamente pelo Painel de Recomendações, antes da apresentação e do conhecimento dos resultados.

A recomendação clínica foi estabelecida de forma consensual em uma reunião de um Painel de Recomendação composto por especialistas indicados pelo patrocinador. Todo o projeto foi supervisionado e financiado pela SBC, enquanto a elaboração da revisão sistemática e o processo de recomendação clínica foram conduzidos por uma equipe independente de metodologistas (LL, AB e QD). A equipe de metodologistas e os autores declaram não possuir nenhum conflito de interesse relevante para esta revisão.

## Resultados

Na busca inicial por revisão sistemática, foram identificadas 815 publicações. Após a exclusão de duplicatas, a triagem por título e resumo, e a aplicação dos critérios de elegibilidade, foram selecionados 17 artigos para leitura completa. Destes, nenhuma revisão respondia especificamente à questão científica proposta, mas dois documentos apresentavam potencial como fonte de referências de artigos sobre atenolol vs outras classes de anti-hipertensivos no tratamento da HA^24,25^ (Figura S1 – Fluxograma PRISMA da seleção de revisões sistemáticas).

Desses dois estudos, a revisão de Wiysonge et al.^24^ foi escolhida como referência para os estudos primários e como base para a estratégia de busca, por ser o documento mais recente e abrangente. Esse documento incluía sete artigos primários comparando atenolol a outras classes de anti-hipertensivos, que poderiam ser incluídos na nova revisão. Além disso, sua estratégia de busca foi utilizada como modelo para atualização da procura por novos ECRs publicados a partir de 2017.^24^

A segunda busca nas bases de dados, agora por ECRs, com o objetivo de atualizar a seleção da revisão sistemática de referência, identificou 6.404 registros, dos quais 669 eram duplicatas e 5.727 estudos foram excluídos com base nos títulos e resumos. Ainda, foi realizada uma revisão manual nas referências das revisões sistemáticas selecionadas na primeira busca. Nesta segunda fase, oito estudos foram selecionados para leitura completa, mas nenhum ECR adicional aos sete artigos primários do estudo de Wiysonge et al.^24^ foi incluído na metanálise em rede, com base nos critérios da pergunta PICO (Figura S2 – Fluxograma PRISMA da seleção de ECRs).

Os detalhes das estratégias de busca, os documentos excluídos após a leitura completa e os fluxogramas PRISMA estão descritos no material suplementar (Tabelas S1 a S4, Figuras S1 e S2).

A nova busca não resultou em estudos adicionais com a intervenção específica (atenolol), e as principais características dos sete ECRs incluídos (selecionados originalmente na revisão de Wiysonge et al.^24^) estão descritas nas Tabelas 1 e 2.

Os estudos analisaram populações de pacientes com HA e diferentes perfis clínicos, com número de participantes variando de 884 a 22.576 e idades médias entre 56 e 79 anos. As populações eram compostas por indivíduos com hipertrofia ventricular esquerda, diabetes melito tipo 2 e fatores de risco cardiovascular adicionais, além de HA. Os demais detalhes da metodologia e do processo de busca/seleção dos estudos podem ser consultados no material suplementar deste estudo.

Os sumários de efeitos são apresentados nos *forest plot*, que comparam diferentes tratamentos à utilização do atenolol (utilizado como referência). São apresentadas as razões de chance (*odds ratio*, OR) e os IC 95%. Valores de OR maiores que 1 indicam que o atenolol apresenta maiores chances de eventos cardiovasculares em comparação ao outro tratamento. Valores de OR menores que 1 indicam menor chance de eventos cardiovasculares em relação ao outro tratamento.

**Figura 1:**
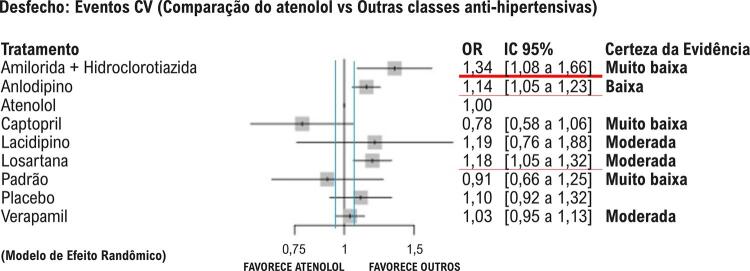
– Desfechos compostos: eventos cardiovasculares maiores (morte, acidente vascular cerebral e infarto agudo do miocárdio). Linhas horizontais vermelhas destacam os estudos com diferenças estatisticamente significativas (linha vermelha fina) e clinicamente relevantes (linha vermelha grossa). As linhas verticais azuis indicam o limiar de diferença mínima importante de 5%. A certeza da evidência para cada comparação é apresentada à direita.

Em relação ao desfecho primário de eventos cardiovasculares maiores, a combinação de amilorida com hidroclorotiazida (HCTZ) mostrou um perfil protetor, caracterizado por menor número de eventos cardiovasculares quando comparada ao atenolol, com resultado acima do limiar de 5% de DMI. Anlodipino e losartana também apresentaram menor número de eventos em relação ao atenolol, embora o limite inferior do IC 95% tenha atingido o limiar de DMI de 5%, o que reduz a confiança em sua relevância clínica (Figura 1).

**Figura 2:**
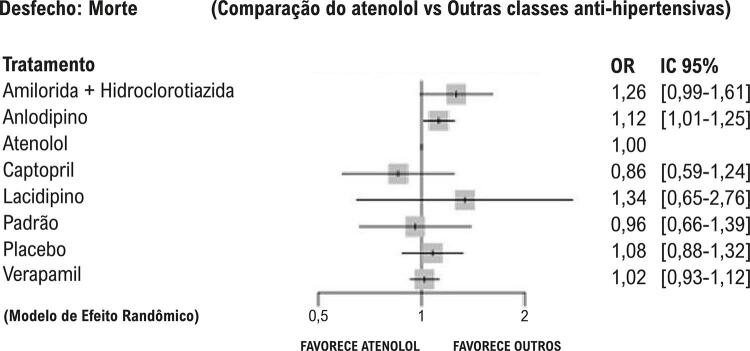
– Desfecho: morte por todas as causas, de acordo com os diferentes tratamentos anti-hipertensivos comparados ao atenolol.

**Figura 3:**
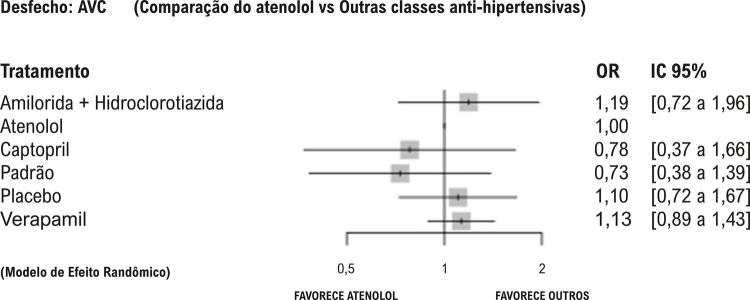
– Desfecho: acidente vascular cerebral, de acordo com os diferentes tratamentos anti-hipertensivos comparados ao atenolol.

**Figura 4:**
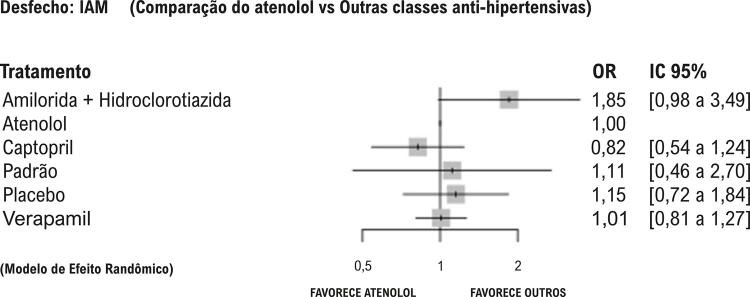
– Desfecho: infarto do miocárdio, de acordo com os diferentes tratamentos anti-hipertensivos comparados ao atenolol.

Para os desfechos secundários — morte (Figura 2), AVC (Figura 3) e IAM (Figura 4) — os IC 95% das comparações ultrapassam o limiar de DMI estabelecido, indicando resultados dentro da margem de não relevância clínica e, em quase sua totalidade, também a linha de efeito neutro, sugerindo ausência de diferença estatisticamente significativa para essas estratégias.

**Figura 5:**
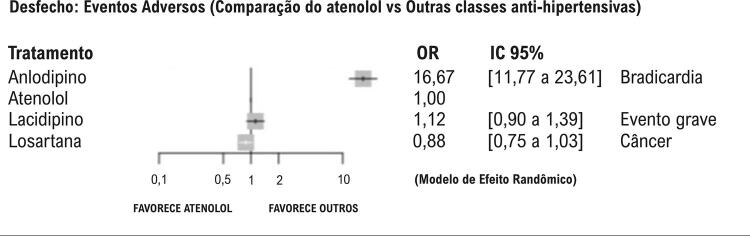
– Desfecho: eventos adversos, de acordo com os diferentes tratamentos anti-hipertensivos comparados ao atenolol.

Apenas três dos sete estudos relataram eventos adversos (Figura 5), sendo que a comparação com anlodipino foi a única que apresentou diferença estatisticamente significativa, com bradicardia mais frequente no grupo do atenolol (OR 16,67; IC 95%: 11,77 a 23,61) — uma consequência clínica já esperada para BB.

**Figura 6:**
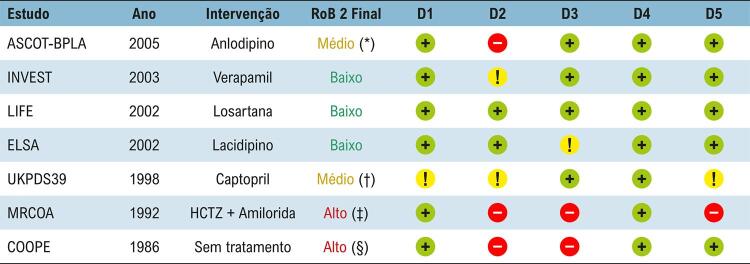
– Risco de viés (RoB 2) dos estudos primários incluídos nesta revisão sistemática. São apresentados os cinco domínios avaliados, com atribuição das cores: verde para baixo risco de viés, amarelo para risco moderado e vermelho para alto risco de viés. Também é mostrado o julgamento final por estudo, com respectivas legendas indicando a característica específica pela qual houve penalização. Desvios no tratamento não explicados (*); Problemas no cegamento, múltiplos comparadores (†); Desvios no tratamento, perda de seguimento e ausência de protocolo (‡); Desvios no tratamento, ausência de informações sobre dados faltantes (§). D1: Randomização; D2: Desvios no tratamento, D3: Dados faltantes; D4: Medição do desfecho; D5: Dados reportados.

O risco de viés e a certeza da evidência foram avaliados para o desfecho primário composto e para os desfechos secundários individuais de morte por todas as causas, IAM e AVC, nos sete ECRs. Na avaliação de risco de viés, são analisados cinco domínios por estudo: randomização, desvios no tratamento, dados faltantes, medida do desfecho e dados reportados. Penalizações aos estudos quanto ao risco de viés ocorreram principalmente por desvios no tratamento (D2) e dados faltantes (D3), como nos estudos MRCOA^26^ e COOPE,^27^ classificados como de alto risco de viés. Os estudos ASCOT-BPLA^28^ e UKPDS39^29^ apresentaram risco de viés moderado devido a problemas no cegamento e desvios não explicados. Os estudos INVEST,^30^ LIFE^31^ e ELSA^32^ foram classificados como de baixo risco de viés (Figura 6 e Material Suplementar).

**Figura 7:**
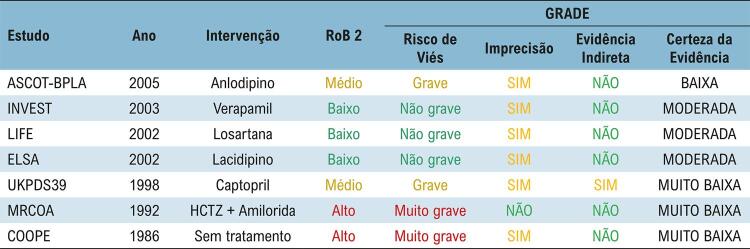
– Classificação GRADE dos estudos primários incluídos nesta revisão sistemática. HCTZ: hidroclorotiazida.

A certeza da evidência, avaliada pelo sistema GRADE, variou de muito baixa a moderada. Nos estudos cujo comparador foi verapamil, losartana ou lacidipino, a certeza da evidência foi classificada como moderada, enquanto nos artigos que compararam hidroclorotiazida com amilorida ou ausência de tratamento, a certeza da evidência foi definida como muito baixa (Figura 7).

**Figura 8:**
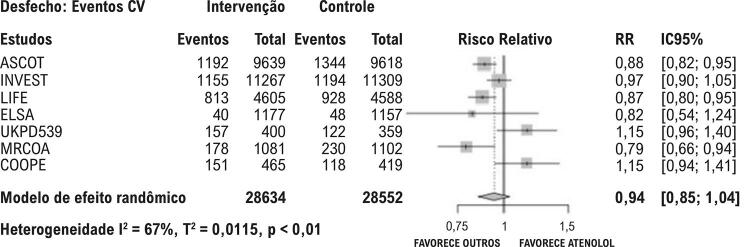
– Metanálise convencional comparando o desfecho composto de eventos cardiovasculares entre o atenolol e os demais anti-hipertensivos agrupados como comparador.

Como análise de sensibilidade, em comparação à metodologia da metanálise em rede, elaborou-se o *forest plot* de uma metanálise convencional, comparando o atenolol às diferentes classes de anti-hipertensivos agrupadas como comparador. Deve-se observar a diferença numérica na estimativa de efeito para cada comparação (atenolol vs demais anti-hipertensivos) entre os resultados da metanálise em rede e os da metanálise convencional. Nesta última, em que as demais classes foram agrupadas, o resultado apresenta grande heterogeneidade (Figura 8), o que corrobora a ideia de que não se deve agrupar classes de anti-hipertensivos com mecanismos de ação tão distintos em um único grupo dentro de uma metanálise.

Para melhor entendimento dos *forest plots* abaixo, que representam os efeitos de cada estratégia medicamentosa, utilizou-se a nomenclatura “padrão” para o braço comparador do estudo COOPE (1998), no qual se comparou atenolol (com possibilidade posterior de adição de bendrofluazida e metildopa, se necessário) à ausência de tratamento. Nesse estudo, o braço comparador só receberia tratamento anti-hipertensivo caso o indivíduo mantivesse valores de PA acima de 280/120 mmHg ou sofresse um AVC. A estratégia nomeada “placebo” refere-se ao terceiro braço do estudo MRCOA (1992), no qual os pacientes foram alocados para uso de atenolol, hidroclorotiazida + amilorida ou placebo.

## Discussão

Os BB constituem uma classe de medicamentos amplamente utilizados no tratamento de doenças cardiovasculares, como HA, insuficiência cardíaca, doença arterial coronariana e arritmias, sendo também indicados para outras condições, como profilaxia de enxaquecas, tremor essencial e controle de sintomas de ansiedade.^24,33,34^ Esses medicamentos atuam por meio do bloqueio dos receptores beta-adrenérgicos, reduzindo os efeitos das catecolaminas no coração e no sistema vascular.^6^ Agem ainda no sistema nervoso central e inibem a secreção de renina pelo aparelho justaglomerular renal.^3^ Existem diferentes subclasses de BB, como os seletivos para receptores β_1_, que possuem menor impacto sobre os receptores β_2_ (p. ex., atenolol, metoprolol e bisoprolol); e os não seletivos, que bloqueiam tanto os receptores β_1_ quanto os β_2_ (p. ex., propranolol). Além disso, alguns BB apresentam propriedades adicionais, como atividade vasodilatadora, observada no carvedilol e no nebivolol.^35^ Entre os efeitos colaterais frequentemente observados, destacam-se bradicardia, fadiga, hipotensão, broncoespasmo (particularmente com os não seletivos) e disfunção sexual.^24^

Nas décadas de 1970 e 1980, esses medicamentos foram considerados de primeira escolha para o tratamento da HA, sendo recomendados, em 1983, pela Organização Mundial da Saúde em conjunto com a Sociedade Internacional de Hipertensão, e, em 1988, pelo *Joint National Committee*.^7,8^ No entanto, especialmente a partir dos anos 1990, foram publicados estudos originais e metanálises comparando os BB a outras classes de medicamentos anti-hipertensivos, questionando o papel desses fármacos no tratamento da HA.

Em 2005, foi publicada uma revisão sistemática incluindo 13 ECRs com 105.951 participantes, comparando os BB, como grupo, a outras classes de anti-hipertensivos. Nesse mesmo documento, foram incluídos sete estudos com 27.433 indivíduos que realizaram a comparação com placebo.^10^ A conclusão afirmava categoricamente que o efeito dos BB era inferior, com risco aumentado de AVC, e, portanto, não deveriam permanecer como primeira escolha no tratamento da HA primária. Neste artigo, os estudos primários incluíram diferentes BB, que foram agrupados e comparados a classes distintas de medicamentos anti-hipertensivos, também agrupadas, além de placebo. Ocasionalmente, o braço de um mesmo estudo poderia utilizar diferentes opções de BB. No sumário final da metanálise, não houve diferença estatisticamente significativa entre os grupos para os desfechos de mortalidade por todas as causas e IAM, mas foi constatada diferença estatisticamente significativa para AVC — embora clinicamente não relevante, pois o IC 95% ultrapassou o limiar de DMI de 5%. Houve uma análise secundária para a comparação exclusiva do atenolol com outras classes de anti-hipertensivos agrupadas, e, nessa avaliação, observou-se diferença estatisticamente significativa para mortalidade e diferença clinicamente relevante em relação ao AVC, ambas a favor das outras classes farmacológicas agrupadas. Não houve avaliação da qualidade dos estudos incluídos.

Em uma revisão sistemática publicada em 2007, Dahlöf et al.^36^ realizaram uma metanálise de estudos com atenolol e outros BB vs placebo ou nenhum tratamento. Naquele momento, os BB já não eram recomendados como opções de primeira escolha para HA. O resultado para o desfecho AVC mostrou uma proteção estatisticamente significativa a favor do atenolol, sem diferença significativa para o desfecho composto de eventos cardiovasculares. Na discussão, os autores destacam que o uso de BB reduz o risco cardiovascular na HA em comparação com placebo ou nenhum tratamento. No entanto, apontam uma clara superioridade a favor da losartana e da anlodipino em estudos recentes da época — LIFE e ASCOT, respectivamente — no desfecho AVC, tornando os BB inadequados como comparadores em novos ECRs.

Até então, não era prática comum avaliar a qualidade dos estudos primários, baseando-se as conclusões apenas nos efeitos numéricos de benefício e malefício de cada estratégia. Um segundo ponto de incerteza metodológica nesses estudos diz respeito à comparação dos BB, de forma geral, com classes distintas de anti-hipertensivos agrupadas, uma vez que bloqueadores dos canais de cálcio, diuréticos tiazídicos e similares, e bloqueadores do sistema renina-angiotensina-aldosterona (SRAA) são medicamentos com mecanismos de ação, perfis de efeitos colaterais e, possivelmente, resultados de eficácia muito distintos.

Em 2017, a Colaboração Cochrane publicou uma revisão sistemática sobre os BB,^24^ analisados também de forma agrupada, mas agora comparados a placebo e a outras classes de anti-hipertensivos avaliadas separadamente: diuréticos, bloqueadores dos canais de cálcio, inibidores do SRAA e alfabloqueadores. Este artigo utilizou a ferramenta GRADE para a análise da certeza da evidência de cada comparação, incluindo a qualidade dos estudos na interpretação final dos dados. Os principais resultados mostraram uma redução estatisticamente significativa de AVC e do desfecho composto de eventos cardiovasculares a favor dos BB, quando comparados ao placebo, mas com IC 95% ultrapassando o limiar de relevância clínica mínima de 5%. Esse cenário foi classificado como de baixa certeza da evidência. Além disso, ao comparar os BB de forma agrupada com os bloqueadores de canais de cálcio (nos desfechos AVC e eventos cardiovasculares) e com os inibidores do SRAA (no desfecho AVC), esses outros medicamentos mostraram redução de eventos clinicamente relevantes, com certeza da evidência graduada como moderada. Assim, permanece o questionamento sobre se o efeito dos BB na PA e nos desfechos clínicos, quando analisados de forma agrupada, seria realmente homogêneo — caracterizando um efeito de classe — ou, como já sugerido, se haveria uma ação distinta entre as diferentes gerações de BB, o que tornaria inadequado agrupá-los em uma única análise.

Em 2020, Thomopoulos et al.^11^ propuseram uma revisão sistemática sobre BB no tratamento da HA. Apesar da inclusão de 87 estudos, apenas 16 eram específicos para HA. Destes, sete avaliaram o atenolol como tratamento ativo, quatro investigaram o propranolol, dois analisaram o metoprolol, um testou o efeito do oxprenolol, um incluiu atenolol ou metoprolol no mesmo braço de intervenção, e um último avaliou qualquer BB. Nessa revisão sistemática, a grande maioria dos ECRs incluídos abordava outras doenças, principalmente doença arterial coronariana e insuficiência cardíaca. A conclusão foi que “em comparação com outros medicamentos anti-hipertensivos, os BB parecem ser substancialmente menos eficazes na proteção contra AVC e mortalidade geral”. O resultado da comparação entre os BB agrupados e as várias classes de anti-hipertensivos também agrupadas mostrou uma diferença clinicamente relevante no desfecho AVC em favor dos outros medicamentos, nos estudos específicos para HA (RR = 1,21; IC 95%: 1,07 a 1,38), e uma diferença clinicamente irrelevante para mortalidade total (RR = 1,06; IC 95%: 1,01 a 1,12). Não houve análise separada para o atenolol, e a heterogeneidade não foi apresentada junto às metanálises nos *forest plots*. A avaliação do risco de viés foi realizada segundo a ferramenta RoB 2, mas os demais componentes do GRADE — heterogeneidade, evidência indireta, imprecisão e viés de publicação — não foram considerados em conjunto para determinar a qualidade do corpo de evidências (certeza da evidência).

Em uma revisão sistemática da Colaboração Cochrane publicada em 2023,^37^ que comparou diuréticos com outras classes de anti-hipertensivos, não foram observadas diferenças nos desfechos avaliados em comparação com os BB — incluindo eventos cardiovasculares, mortalidade total, AVC, IAM e insuficiência cardíaca. A certeza da evidência variou entre baixa e moderada.

Apesar desses resultados, com conclusões distintas — algumas neutras e outras desfavoráveis aos BB —, observa-se que essa classe de medicamentos continua sendo amplamente utilizada na prática clínica para o tratamento da HA. Em um estudo transversal publicado em 2024 por Prejbisz et al.,^38^ envolvendo médicos da Itália, Polônia e Turquia, cerca de 23% dos profissionais relataram prescrever BB para o tratamento da HA, proporção que aumentava para 30% quando havia comorbidades cardiovasculares concomitantes.

A presente revisão sistemática foi elaborada com base em algumas premissas metodológicas. Primeiro, o efeito anti-hipertensivo das diferentes substâncias da classe dos BB parece não ser homogêneo, pois alguns atuam apenas sobre os receptores β_1_, outros sobre os receptores β_1_ e β_2_, e ainda há aqueles com efeitos vasodilatadores.^6^ Portanto, agrupá-los em um único conjunto, assumindo um mesmo efeito anti-hipertensivo, pode não ser o ideal. Em segundo lugar, agrupar diversas classes de medicamentos anti-hipertensivos como um comparador único aumenta significativamente a heterogeneidade entre os estudos, reduzindo de forma relevante a confiança na estimativa do efeito. Em terceiro lugar, devemos sempre basear nossas conclusões na estimativa do efeito comparativo (razão de risco), considerando a DMI previamente estabelecida, a fim de determinar se uma estratégia gera um efeito clinicamente relevante. E, por fim, é fundamental associar a estimativa de efeito à certeza da evidência atribuída a cada estudo que sustenta esse resultado, preferencialmente por meio da ferramenta GRADE — que determina o grau de confiança que temos naquela informação. Se essa qualidade for baixa ou muito baixa, é possível que, na prática, a estimativa de efeito seja consideravelmente diferente da observada, o que reforça a necessidade de novos estudos clínicos para sustentar qualquer conclusão confiável.^39^

Diante dessas premissas, e com o objetivo de preencher a lacuna de conhecimento existente, decidiu-se comparar o atenolol isoladamente — considerado o BB mais prescrito no tratamento da HA no Brasil — com outras classes de anti-hipertensivos não agrupadas. De forma surpreendente, verificou-se o número limitado de estudos que avaliaram essa comparação direta, sendo que nenhum dos medicamentos comparadores foi repetido em mais de um estudo original. No geral, são estudos concluídos há mais de 20 anos, com qualidade metodológica bastante variável e resultados considerados imprecisos.

A metodologia de metanálise em rede empregada neste documento, apesar de, neste caso, não contemplar comparações indiretas ou em rede, apresenta a vantagem de estimar o efeito do atenolol com maior precisão, sem agrupar as diversas classes distintas de anti-hipertensivos, como ocorre em uma metanálise tradicional. Além disso, não foi elaborado um ranking entre as diferentes classes de anti-hipertensivos — ferramenta de hierarquização disponível nessa metodologia — devido à grande variabilidade na certeza da evidência dos estudos originais.

Entre as comparações que apresentaram vantagem no desfecho composto de “eventos cardiovasculares” a favor de outras classes anti-hipertensivas, o maior efeito (hidroclorotiazida com amilorida) foi associado a uma certeza da evidência muito baixa — o que sugere que o verdadeiro resultado pode ser substancialmente diferente do observado. Para as outras duas classes que mostraram benefício (anlodipino e losartana), o intervalo de confiança toca o limiar da DMI previamente estabelecida, com certeza da evidência classificada como baixa e moderada, respectivamente.

Vale destacar que algumas recomendações de sociedades médicas contrárias ao uso do atenolol são baseadas em fatores como potência, dose, frequência de uso e interações farmacocinéticas. Além disso, doses de 100 a 200 mg por dia mostraram-se mais eficazes do que doses de 25 e 50 mg por dia em pacientes com angina, o que pode também se aplicar ao tratamento da HA.^40,41^

Um fato que parece não deixar dúvidas é a intensidade da redução da PA nas combinações que utilizaram o atenolol como medicamento inicial, em comparação às combinações administradas no braço comparador. Em todos os estudos originais incluídos, a redução da PA foi bastante semelhante entre o atenolol e os medicamentos comparados, sugerindo um efeito na redução da PA similar ao das demais classes de anti-hipertensivos.

Este documento apresenta algumas limitações. A primeira, e mais importante, refere-se ao número reduzido de estudos com comparações diretas entre atenolol e outras classes de anti-hipertensivos. Para responder adequadamente à pergunta PICO apresentada, seriam necessários, idealmente, mais estudos, com publicações mais recentes e boa qualidade metodológica. Essa limitação se aplica, de forma ainda mais acentuada, aos estudos em HA com outros BB, como metoprolol, bisoprolol, carvedilol e nebivolol, para os quais há uma escassez ainda maior de artigos, impossibilitando uma análise fidedigna. Em segundo lugar, apesar da elaboração de uma metanálise em rede, a ausência de comparações indiretas no tema impossibilitou a realização de comparações em rede. Em terceiro lugar, o BB não foi utilizado como monoterapia nos estudos incluídos (Tabela 2). Esse aspecto reflete a prática clínica habitual no tratamento da HA, em que a associação de medicamentos é frequentemente necessária para alcançar as metas recomendadas. No entanto, essa característica limita a interpretação sobre o efeito anti-hipertensivo observado, dificultando a distinção entre o que pode ser atribuído exclusivamente ao atenolol e o que resulta da coadministração de outras medicações. Esse mesmo raciocínio se aplica aos medicamentos comparadores, que também não foram utilizados isoladamente nos estudos. Considerando a baixa eficácia da monoterapia no controle da HA na maioria dos pacientes, é pouco provável que se realizem estudos clínicos capazes de isolar o efeito de um único medicamento de forma precisa.

**Tabela 2:** – Características dos ensaios clínicos incluídos nesta revisão sistemática (destacando as medicações anti-hipertensivas coadministradas em cada estudo)

Estudo	Ano	Intervenção	Anti-hipertensivos adicionais	Desfechos primários	Desfechos secundários
ASCOT-BPLA	2005	Anlodipino	No grupo anlodipino: perindopril; no grupo atenolol: bendrofluazida e potássio	IAM fatal e não fatal	Morte, AVC, eventos coronarianos, eventos CV, procedimentos cardiovasculares, insuficiência cardíaca
INVEST	2003	Verapamil	Trandolapril e/ou hidroclorotiazida	Morte, IAM não fatal e AVC não fatal	Morte cardiovascular, angina, eventos adversos, hospitalização e controle pressórico no 24º mês
LIFE	2002	Losartana	Hidroclorotiazida e anti-hipertensivos de outras classes	Evento CV	Morte, hospitalização por insuficiência cardíaca
ELSA	2002	Lacidipino	Hidroclorotiazida	EMI	Aumento ou redução da placa carotídea (EMI ≥ 1,3 mm), morte cardiovascular e evento cardiovascular não fatal
UKPDS39	1998	Captopril	Furosemida, nifedipino, metildopa, prazosina	Morte e morte relacionada ao DM2	Complicações macro e microvasculares, morte, albuminúria, retinopatia, IAM, DAC, DAOP, amputação
MRCOA	1992	Hidroclorotiazida + Amilorida	Se necessário, a intervenção do outro braço era utilizada; também nifedipino	Morte, AVC, eventos coronarianos	NA
COOPE	1986	Sem tratamento	Bendrofluazida e alfa-metildopa	Morte, AVC, IAM	Sintomas

AVC: acidente vascular cerebral; CV: cardiovascular; DM2: diabetes melito tipo 2; DAC: doença arterial coronariana; DAOP: doença arterial obstrutiva periférica; EMI: espessura média intimal; IAM: infarto agudo do miocárdico; NA: não se aplica.

Esses fatos impõem um obstáculo adicional à formulação de uma Recomendação Clínica sobre a pergunta PICO proposta — uma resposta tão necessária, considerando que a utilização do atenolol no tratamento da HA é uma realidade.^42^

Os membros do painel desta Recomendação Clínica da SBC entendem que a interpretação da literatura prévia sobre o tema ocorreu em um período em que a avaliação da qualidade dos estudos primários ainda era pouco utilizada, o que pode ter contribuído para uma supervalorização das diferenças entre os BB e outras classes de anti-hipertensivos nos principais desfechos cardiovasculares. Ao incorporar o grau de certeza da evidência, concluiu-se que a diferença entre o atenolol e outras classes de anti-hipertensivos pode ser pequena, e que as reduções nos valores de PA são similares.

## Conclusão

A HA é uma condição altamente prevalente na população adulta, e sua prevenção e tratamento devem ser prioridades para qualquer sistema de saúde. Os achados desta revisão enfatizam a necessidade frequente da associação de anti-hipertensivos no esquema terapêutico, a fim de alcançar metas que previnam lesões de órgãos-alvo e desfechos adversos.

Apesar das evidências limitadas, o atenolol demonstrou eficácia comparável na redução da PA, com pequenas diferenças nos desfechos cardiovasculares a favor de outras classes de anti-hipertensivos. A recomendação deste painel de especialistas é que o atenolol pode ser considerado entre as opções para associação medicamentosa no tratamento da HA primária em adultos. Outros BBs não foram avaliados nesta revisão sistemática.

Como em qualquer recomendação clínica, sugere-se que a prescrição seja individualizada, considerando as comorbidades, características clínicas e preferências de cada paciente, além do julgamento clínico do profissional prescritor.

**Tabela 1:** – Características dos ensaios clínicos incluídos nesta revisão sistemática

Estudo	Ano	Intervenção	Seguimento (anos)	População	Idade (média)	Pacientes	PA média inicial (mmHg)	PA média final no grupo de tratamento (mmHg)	PA média final no grupo atenolol (mmHg)
ASCOT-BPLA (†)	2005	Anlodipino	5,5	HA e ≥3 FRCV	40 a 79 (63)	19.257	164/94	136/77	137/79
INVEST	2003	Verapamil	2,7	HA e DAC, >50 anos	≥50 (66)	22.576	150/87 (//)	131,3/77	131/76,8
LIFE (*)	2002	Losartana	4,8	HA e HVE	55 a 80	9.193	174/97	143,8/80,4	144,9/80,6
ELSA (‡)	2002	Lacidipino	3,75	HA	45 a 75 (56)	2.334	163/101	141,4/85,5	141,2/85,4
UKPDS39	1998	Captopril	8,4	HA + DM2	26 a 65 (56)	1.148	160/94	144/83	143/81
MRCOA	1992	HCTZ + Amilorida	5,8	HA	65 a 74 (70)	4.396	183/91	150/77	150/77
COOPE (§)	1986	Sem tratamento	4,4	HA	60 a 79 (65)	884	196/99	180/89	178/88

(*) Eventos CV = morte total; morte CV + IAM fatal e não fatal; AVC fatal e não fatal. (†) Não está claramente definido se os resultados de IAM e AVC incluem apenas os não fatais ou também os fatais. (‡) Eventos CV definidos como IAM, AVC e morte CV apenas (exclui morte total). (§) Eventos CV = morte total, AVC total e doença coronariana total. (//) Média ponderada da PA entre pacientes que usavam e não usavam anti-HA. CV: cardiovascular; HA: hipertensão arterial; FRCV: fatores de risco cardiovascular; DAC: doença arterial coronariana; IAM: infarto agudo do miocárdio; HVE: hipertrofia ventricular esquerda; DM2: diabetes melito tipo 2; PA: pressão arterial; anti-HA: anti-hipertensivos; HCTZ: hidroclorotiazida.
