# Supplementary material for: Revisão Sistemática Sobre a Eficácia do Atenolol no Tratamento Anti-Hipertensivo: Recomendação da Sociedade Brasileira de Cardiologia
Source: Arq Bras Cardiol. 2025 Sep 8;122(9):e20250034. [Article in Portuguese] doi: 10.36660/abc.20250034 (PMC12677729; doi:10.36660/abc.20250034)
Supplement: Supplementary file 1 [file 0066-782x-abc-122-09-e20250034-suppl01.pdf]

Material Suplementar

Página 1: Detalhes da metodologia da revisão sistemática rápida.

Página 3: Tabela S1 - Estratégia de busca por revisões sistemáticas.

Página 7: Figura S1 – Fluxograma PRISMA da seleção de revisões sistemáticas.

Página 8: Tabela S2 - Estratégia de busca por revisões sistemáticas.

Página 14: Figura S2 – Fluxograma PRISMA da seleção de ensaios clínicos randomizados.

Página 15: Tabela S3 - revisões sistemáticas excluídas na seleção por texto completo e motivos da exclusão.

Página 17: Tabela S4 - Ensaios clínicos excluídos na etapa de leitura do texto completo e motivos da exclusão.

Página 18: Tabela S5 - PRISMA da revisão sistemática.

1. Detalhes da metodologia da revisão sistemática rápida.

Com base na pergunta estruturada e de acordo com a estratégia PICO, foram realizadas buscas nas bases de dados MEDLINE via Pubmed, EMBASE e na Colaboração Cochrane. A busca por revisões sistemáticas foi realizada em 17 de junho de 2024.

As estratégias de busca foram elaboradas de acordo com o vocabulário controlado de cada uma das bases pesquisadas e incluíram apenas descritores relacionados à doença e às intervenções a fim de aumentar a sensibilidade da busca. Foi utilizado filtro de tempo (publicação desde 2013) e não foram utilizados limites de idioma. A triagem dos estudos foi realizada com o *software* Rayyan por dois investigadores independentes e as divergências resolvidas por consenso, em duas etapas. Na primeira etapa, foi realizada a leitura dos títulos e resumos das referências identificadas, sendo os estudos potencialmente elegíveis pré-selecionados. Na segunda etapa, foi realizada leitura do texto completo para confirmação da elegibilidade e seleção final.

Na busca inicial por revisões sistemáticas, foram identificadas 815 publicações. Após a exclusão de duplicatas e a triagem por título e resumo, foram selecionados 17 estudos para leitura completa, conforme fluxograma PRISMA (Figura S1)

Ao final da seleção, foram evidenciadas duas revisões sistemáticas: 1) o estudo de Kuyper e colaboradores publicado em 2014 (Kuyper L.) que incluiu apenas duas bases de

dados, sendo, portanto, excluído; e 2) a revisão sistemática de Wiysonge e colaboradores publicada em 2017, que incluiu a busca em três bases de dados. Esta revisão foi incluída em nossas análises, porém foi necessária atualizá-la. A busca realizada na revisão de Wiysonge et al (2017) foi adaptada e realizada nas três bases de dados, sendo utilizado filtro de tempo para incluir estudos a partir de 2015 [ano em que a busca de Wiysonge et al (2017) foi finalizada].

Esta nova busca, com a intervenção específica (atenolol), não resultou em nenhum estudo.

Para a extração de dados dos estudos originais, um pesquisador realizou a coleta de todas as variáveis pré-definidas em uma planilha estruturada. Os dados de efeito foram extraídos independentemente por dois pesquisadores e incluíram as características basais de cada estudo e desfechos de eficácia (mortalidade por todas as causas, AVC e IM, além de desfechos de segurança, quando descritos. Divergências foram resolvidas em consenso.

Os dados extraídos foram analisados utilizando odds ratio (OR) com seus respectivos intervalos de confiança de 95% (IC 95%),_comparando o efeito entre o atenolol e as demais intervenções avaliadas através de uma meta-análise em rede. O modelo bayesiano foi adotado e o ranking probabilístico não foi incluído no documento principal pela grande variabilidade da qualidade dos estudos analisados (de muito baixa a moderada). Os desfechos foram apresentados em gráficos de floresta para facilitar a visualização dos resultados. Para os desfechos de segurança, os eventos adversos relatados foram incluídos na análise sempre que disponíveis.

A qualidade metodológica das revisões sistemáticas incluídas foi avaliada pelos dois pesquisadores, independentemente, usando a ferramenta RoB 2 (*Risk of Bias* 2), produzida pela organização Cochrane. A ferramenta permite avaliar o viés no processo de randomização, desvios da intervenção pretendida, viés por dados faltantes, na aferição e no relato dos desfechos. A estrutura GRADE (*Grading of Recommendations, Assessment, Development and Evaluations*) foi usada para avaliar a qualidade da evidência e determinar a força das recomendações, quando viável.

Tabela S1 - Estratégia de busca por revisões sistemáticas.

Data	Base	Busca	Total
06/17/2024	Medline	"phenoxypropanolamines"[MeSH Terms] OR “phenoxypropanolamines”[Title/Abstract] OR “Phenoxy-Propanolamines”[Title/Abstract] OR “Phenoxy Propanolamines”[Title/Abstract] OR “Atenolol"[MeSH Terms] OR “Atenolol”[Title/Abstract] OR “Tenormine”[Title/Abstract] OR “Tenormin”[Title/Abstract] OR “ICI-66082”[Title/Abstract] OR “ICI66082”[Title/Abstract] OR “ICI 66082”[Title/Abstract] OR “Bisoprolol"[MeSH Terms] OR “Bisoprolol”[Title/Abstract] OR “Concor”[Title/Abstract] OR “Bisoprolol Fumarate”[Title/Abstract] OR “Fumarate, Bisoprolol”[Title/Abstract] OR “Bisoprolol Fumarate”[Title/Abstract] OR “Hydrochloride, Bisoprolol”[Title/Abstract] OR “EMD-33512”[Title/Abstract] OR “EMD 33512”[Title/Abstract] OR “CL-297939”[Title/Abstract] OR “CL 297939”[Title/Abstract] OR “Metoprolol"[MeSH Terms] OR “Metoprolol”[Title/Abstract] OR “Betaloc”[Title/Abstract] OR “Betaloc-Astra”[Title/Abstract] OR “Betaloc Astra”[Title/Abstract] OR “Spesicor”[Title/Abstract] OR “Spesikor”[Title/Abstract] OR “H 93-26”[Title/Abstract] OR “H 93 26”[Title/Abstract] OR “CGP-2175”[Title/Abstract] OR “CGP2175”[Title/Abstract] OR “CGP 2175”[Title/Abstract] OR “Metoprolol Tartrate”[Title/Abstract] OR “Lopressor”[Title/Abstract] OR “Betalok”[Title/Abstract] OR “Beloc-Duriles”[Title/Abstract] OR “Beloc Duriles”[Title/Abstract] OR “Metoprolol Succinate”[Title/Abstract] OR “Metoprolol CR-XL”[Title/Abstract] OR “Metoprolol CR XL”[Title/Abstract] OR “Toprol-XL”[Title/Abstract] OR “Toprol XL”[Title/Abstract] OR “Seloken”[Title/Abstract] OR “Toprol”[Title/Abstract] OR “Propranolol"[MeSH Terms] OR “Propranolol”[Title/Abstract] OR “Propanolol”[Title/Abstract] OR “AY-20694”[Title/Abstract] OR “AY 20694”[Title/Abstract] OR “Propranolol Hydrochloride”[Title/Abstract] OR “Hydrochloride, Propranolol”[Title/Abstract] OR “Inderal”[Title/Abstract] OR “Avlocardyl”[Title/Abstract] OR “Obsidan”[Title/Abstract] OR “Obzidan”[Title/Abstract] OR “Dociton”[Title/Abstract] OR “Betadren”[Title/Abstract] OR “Dexpropranolol”[Title/Abstract] OR “Anaprilin”[Title/Abstract] OR “Anapriline”[Title/Abstract] OR “Carvedilol"[MeSH Terms] OR “Carvedilol”[Title/Abstract] OR “Coreg”[Title/Abstract] OR “Dilatrend”[Title/Abstract] OR “Carvedilol Hydrochloride”[Title/Abstract] OR “Eucardic”[Title/Abstract] OR “BM 14190”[Title/Abstract] OR “BM-14190”[Title/Abstract] OR “BM14190”[Title/Abstract] OR “Kredex”[Title/Abstract] OR “Adrenergic beta-1 Receptor Antagonists”[Pharmacological Action]	57

		AND<br>("hypertension" [MeSH Terms] OR "essential hypertension"[MeSH Terms] OR hypertension[Title/Abstract] OR 'acute hypertension'[Title/Abstract] OR 'arterial hypertension'[Title/Abstract] OR 'blood pressure, high'[Title/Abstract] OR 'high blood pressure'[Title/Abstract] OR "essential hypertension"[Title/Abstract] OR 'essential arterial hypertension'[Title/Abstract] OR 'hypertension, essential'[Title/Abstract] OR 'secondary hypertension'[Title/Abstract] OR 'primary hypertension'[Title/Abstract])<br>AND<br>10 years filter	
06/17/2024	Cochrane Library	Propranolol OR Atenolol OR Metoprolol OR Bisoprolol OR Carvedilol OR Fenoxipropanolaminas OR “Adrenergic beta-1 Receptor Antagonists” in Title Abstract Keyword AND "hypertension" OR Essential Hypertension OR arterial hypertension OR high blood pressure OR 'blood pressure, high' OR primary hipertension in Title Abstract Keyword	Cochrane reviews: 21
06/20/2024	EMBASE	('hypertension'/exp OR 'htn (hypertension)' OR 'acute hypertension' OR 'arterial hypertension' OR 'blood pressure, high' OR 'cardiovascular hypertension' OR 'controlled hypertension' OR 'endocrine hypertension' OR 'high blood pressure' OR 'high renin hypertension' OR 'hypertension' OR 'hypertensive disease' OR 'hypertensive effect' OR 'hypertensive reaction' OR 'hypertensive response' OR 'neurogenic hypertension' OR 'preexistent hypertension' OR 'salt high blood pressure' OR 'salt hypertension' OR 'secondary hypertension' OR 'systemic hypertension' OR 'essential hypertension'/exp OR 'essential arterial hypertension' OR 'essential hypertension' OR 'hypertension, essential' OR 'hypertension, idiopathic' OR 'idiopathic hypertension' OR 'primary hypertension' OR 'spontaneous hypertension') AND ('phenoxypropanolamine derivative'/exp OR 'phenoxypropanolamine compound' OR 'phenoxypropanolamine derivative' OR 'phenoxypropanolamine series' OR 'phenoxypropanolamines' OR 'propranolol'/exp OR '1 (1 methylethylamino) 3 (1 naphthyloxy) 2 propanol' OR '1 (1 methylethylamino) 3 (1 naphthyloxy) propan 2 ol' OR '1 (2 hydroxy 3 isopropylaminopropoxy) naphthalene' OR '1 (2 propanylamino) 3 (1 naphthyloxy) propan 2 ol' OR '1 (isopropylamino) 3 (1 naphthyloxy) 2 propanol' OR '1 (propan 2 ylamino) 3 (1 naphthyloxy) propan 2 ol' OR '1 isopropylamino 3 (1 naphthoxy) 2 propanol' OR '1 isopropylamino 3 (1 naphthyloxy) propan 2 ol' OR 'acifol' OR 'adrexan' OR 'alperol' OR 'anaprilin' OR 'anapriline' OR 'anaprilinium' OR 'anapryline' OR 'angilol' OR 'angilol la' OR 'apo-propranolol' OR 'apsolol' OR 'arcablock' OR 'arcablock retard' OR 'artensol' OR 'authus' OR 'avlocardyl' OR 'avlocardyl retard' OR 'ay 64043' OR 'ay64043' OR 'becardin' OR 'bedranol' OR 'beprane' OR 'bercolol' OR 'berkolol' OR 'beta neg' OR 'beta tablinen' OR 'beta tablinen retard' OR 'beta timelets' OR 'beta-timelets' OR 'betabloc' OR 'betadipresan' OR 'betaneg' OR 'betaprol' OR 'betares' OR 'betaryl' OR 'blocard' OR 'blocaryl' OR 'cardinol' OR 'cardinol la' OR 'ciplar' OR 'corbeta' OR 'deralin' OR 'dextrolevo propranolol' OR 'dibudinate' OR 'dideral' OR 'dl propanolol hydrochloride' OR 'dl propranolol' OR 'dociton' OR 'dociton retard' OR 'docitone' OR 'durabeton' OR 'duranol' OR 'efektolol' OR 'efektolol retard' OR 'elbrol' OR 'emforal' OR 'farmadral' OR 'farprolol' OR 'frekven'	737

		OR 'frina' OR 'hemangeol' OR 'hemangiol' OR 'hopranolol' OR 'ici 45520' OR 'ikopal' OR 'impral' OR 'inderal' OR 'inderal la' OR 'inderal retard' OR 'inderalici' OR 'inderex' OR 'indicardin' OR 'indobloc' OR 'innopran' OR 'innopran xl' OR 'inpanol' OR 'ipran' OR 'l propranolol' OR 'lederpronol' OR 'levo propranolol' OR 'levopropranolol' OR 'm 7030' OR 'm7030' OR 'napriline' OR 'noloten' OR 'nsc 91523' OR 'obsidan' OR 'obsin' OR 'obzidan' OR 'oposim' OR 'phanerol' OR 'prandol' OR 'prano puren' OR 'pranopuren' OR 'prestoral' OR 'prolol' OR 'prolol plus' OR 'pronovan' OR 'propabloc' OR 'propal' OR 'propalong' OR 'propanolol hydrochloride' OR 'propayerst' OR 'propercuten' OR 'prophylux' OR 'propra ratiopharm' OR 'propral' OR 'propranolol' OR 'propranolol hydrochloride' OR 'propranolol hydrochloride intensol' OR 'propranolol isomer' OR 'propranur' OR 'proprasylyt' OR 'proprasylyte' OR 'rec 0551' OR 'rec0551' OR 'reducor' OR 'sagittol' OR 'slow deralin' OR 'stapranolol' OR 'sumial' OR 'tenomal' OR 'tensiflex' OR 'waucoton' OR 'atenolol'/exp OR '1 (4 carbamoylmethylphenoxy) 3 isopropylamino 2 propanol' OR '2 [4 [2 hydroxy 3 (isopropylamino) propoxy] phenyl] acetamide' OR '4 (2 hydroxy 3 isopropylaminopropoxy) phenylacetamide' OR 'ablok' OR 'adoll' OR 'alonet' OR 'altol' OR 'angipress (atenolol)' OR 'anolene' OR 'anolpin' OR 'anselol' OR 'apo-atenolol' OR 'arandin' OR 'asten' OR 'atarox' OR 'atcardil' OR 'atecard' OR 'atehexal' OR 'atelol' OR 'atenblock' OR 'atendol' OR 'atenet' OR 'ateni' OR 'atenil' OR 'ateno' OR 'atenogamma' OR 'atenol' OR 'atenolol' OR 'atereal' OR 'aterol' OR 'atestad' OR 'atinol' OR 'atolmin' OR 'b-vasc' OR 'betablok' OR 'betacar' OR 'betarol' OR 'betatop ge' OR 'beten' OR 'bloket' OR 'blokium' OR 'blotex' OR 'cardioten' OR 'catenol' OR 'coratol' OR 'corotenol' OR 'durabeta' OR 'esatenolol' OR 'evitocor' OR 'farnormin' OR 'felo-bits' OR 'hypernol' OR 'hypoten' OR 'ici 66, 082' OR 'ici 66082' OR 'internolol' OR 'lo-ten' OR 'loten' OR 'lotenal' OR 'martenol' OR 'mirobect' OR 'myocord' OR 'neotenol' OR 'nolol' OR 'normalol' OR 'normaten' OR 'normiten' OR 'nortelol' OR 'noten' OR 'oraday' OR 'ormidol' OR 'paesumex' OR 'plenacor' OR 'preloc' OR 'premorine' OR 'prenolol' OR 'prenormine' OR 'ranlol' OR 'rozamin' OR 'serten' OR 'stermin' OR 'temoret' OR 'tenblok' OR 'tenidon' OR 'tenoblock' OR 'tenocor' OR 'tenol' OR 'tenolin' OR 'tenolol' OR 'tenopress' OR 'tenoprin' OR 'tenormin' OR 'tenormin mite' OR 'tenormine' OR 'tenostat' OR 'tensig' OR 'tensinor' OR 'ternolol' OR 'therabloc' OR 'tn 891' OR 'tn891' OR 'tredol' OR 'vascoten' OR 'velorin' OR 'vericordin' OR 'wesipin' OR 'metoprolol'/exp OR '1 isopropylamino 3 [4 (2 methoxyethyl) phenoxy] 2 propanol' OR 'beloc duriles' OR 'belok zok' OR 'betaloc' OR 'h 93-26' OR 'metoprolol' OR 'metoprolol durules' OR 'metoprolol oros' OR 'metropolol' OR 'bisoprolol'/exp OR '1 [[alpha (2 isopropoxyethoxy) para tolyl] oxy] 3 isopropylamino 2 propanol' OR '1 [4 [(2 isopropoxyethoxy) methyl] phenoxy] 3 isopropylamino 2 propanol' OR 'bisoprolol' OR 'carvedilol'/exp OR '1 (4 carbazolyloxy) 3 [2 (2 methoxyphenoxy) ethylamino] 2 propanol' OR '1 (carbazol 4 yloxy) 3 [2 (2 methoxyphenoxy) ethylamino] 2 propanol' OR 'bm 14190' OR 'bm14190' OR 'cardiol (carvedilol)' OR 'cardivas' OR 'carvedilol' OR 'carvedilol phosphate' OR 'carvedlol' OR 'carvipress' OR 'carvrol' OR 'coreg' OR 'coreg cr' OR 'dilatrend' OR 'dilbloc' OR 'dimitone' OR 'dq 2466' OR 'dq2466' OR 'eucardic' OR 'kredex' OR 'querto' OR 'skf 105517' OR 'skf105517' OR 'v-bloc' OR 'adrenergic receptor blocking agent'/exp OR 'adrenergic antagonist' OR 'adrenergic antagonists' OR 'adrenergic block agent' OR 'adrenergic blocker' OR 'adrenergic blocking agent' OR 'adrenergic blocking drug' OR 'adrenergic inhibitor' OR 'adrenergic receptor antagonist' OR 'adrenergic receptor blocker' OR 'adrenergic receptor blocking agent' OR 'adrenolytic' OR 'adrenolytic agent' OR 'antagonist, adrenergic receptor' OR 'antiadrenalin agent' OR 'antiadrenaline agent' OR 'antiadrenergic agent' OR 'antiadrenergic drug' OR 'antiadrenergics' OR 'antiadrenergics, central' OR 'antiadrenergics, peripheral' OR 'sympathetic depressant' OR	

		'sympathicolytic agent' OR 'sympatholytic agent' OR 'sympatholytic drug' OR 'sympatholytica' OR 'sympatholytics' OR 'sympathycolytic agent') AND ('systematic review'/exp OR 'review, systematic' OR 'systematic review' OR 'meta analysis'/exp OR 'analysis, meta' OR 'meta analysis' OR 'meta-analysis' OR 'metaanalysis') (AND [embase]/lim NOT ([embase]/lim AND [medline]/lim)) (AND (2013:py OR 2014:py OR 2015:py OR 2016:py OR 2017:py OR 2018:py OR 2019:py OR 2020:py OR 2021:py OR 2022:py OR 2023:py OR 2024:py)	

Figura S1 – Figura PRISMA da seleção de revisões sistemáticas.

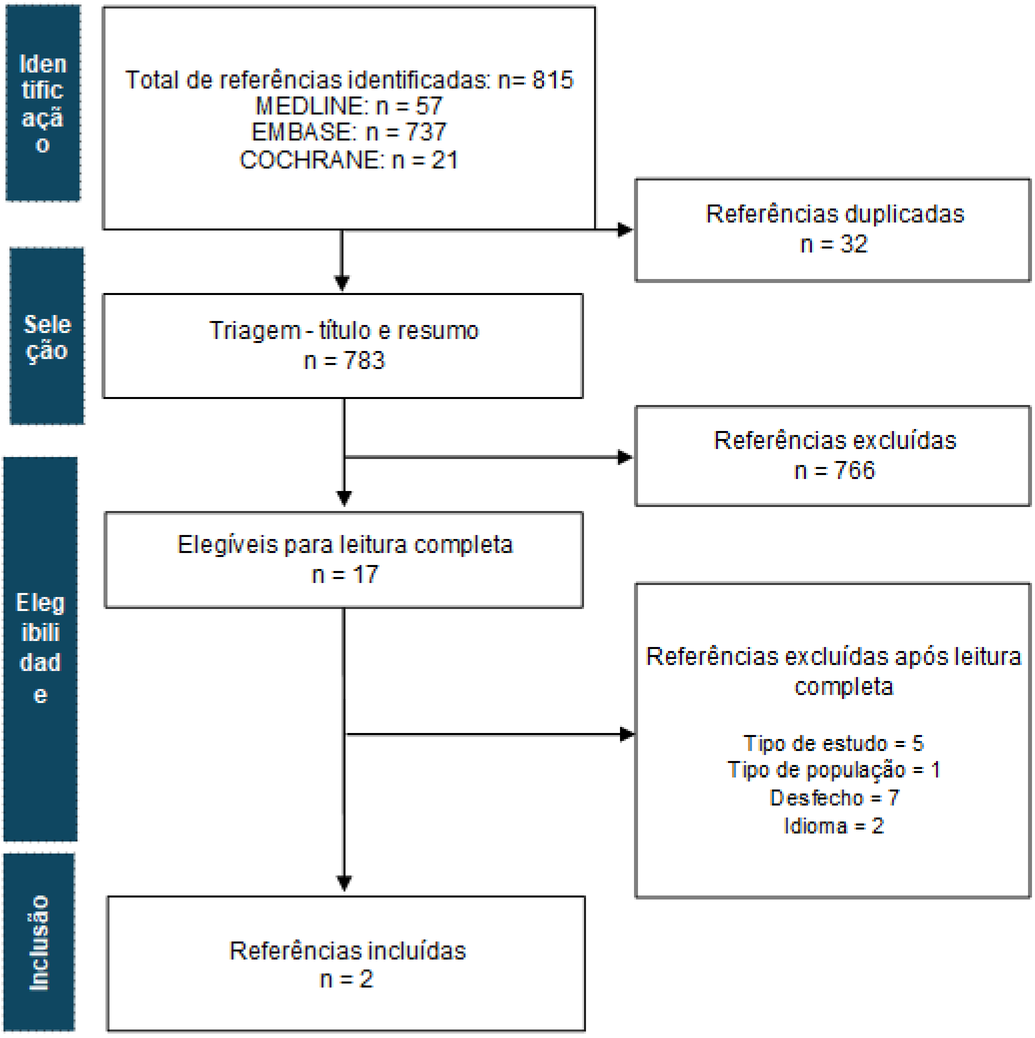

Tabela S2 – Estratégia de busca por ECR para atualização da revisão sistemática.

Pubmed via medline 01/08/2024	#1 adrenergic beta-antagonists[MeSH Terms]<br>#2 "acebutolol"[MeSH Terms] OR "acebutolol"[All Fields] OR "acebutolol s"[All Fields] OR ("adimolol"[Supplementary Concept] OR "adimolol"[All Fields]) OR ("alprenolol"[MeSH Terms] OR "alprenolol"[All Fields]) OR ("amosulalol"[Supplementary Concept] OR "amosulalol"[All Fields]) OR ("arotinolol"[Supplementary Concept] OR "arotinolol"[All Fields]) OR ("atenolol"[MeSH Terms] OR "atenolol"[All Fields]) OR ("befunolol"[Supplementary Concept] OR "befunolol"[All Fields]) OR ("betaxolol"[MeSH Terms] OR "betaxolol"[All Fields]) OR ("bevantolol"[Supplementary Concept] OR "bevantolol"[All Fields]) OR ("bisoprolol"[MeSH Terms] OR "bisoprolol"[All Fields]) OR ("bopindolol"[Supplementary Concept] OR "bopindolol"[All Fields]) OR ("bornaprolol"[Supplementary Concept] OR "bornaprolol"[All Fields]) OR ("bucindolol"[Supplementary Concept] OR "bucindolol"[All Fields]) OR ("bucumolol"[Supplementary Concept] OR "bucumolol"[All Fields]) OR ("bufetolol"[Supplementary Concept] OR "bufetolol"[All Fields]) OR ("bufuralol"[Supplementary Concept] OR "bufuralol"[All Fields]) OR ("bunitrolol"[Supplementary Concept] OR "bunitrolol"[All Fields]) OR ("levobunolol"[MeSH Terms] OR "levobunolol"[All Fields] OR "bunolol"[All Fields] OR "bunolol"[MeSH Terms]) OR ("bupranolol"[MeSH Terms] OR "bupranolol"[All Fields]) OR ("butofilolol"[Supplementary Concept] OR "butofilolol"[All Fields]) OR ("butoxamine"[MeSH Terms] OR "butoxamine"[All Fields]) OR ("carazolol"[Supplementary Concept] OR "carazolol"[All Fields]) OR ("carteolol"[MeSH Terms] OR "carteolol"[All Fields]) OR ("carvedilol"[MeSH Terms] OR "carvedilol"[All Fields] OR "carvedilol s"[All Fields]) OR ("celiprolol"[MeSH Terms] OR "celiprolol"[All Fields]) OR ("cetamolol"[Supplementary Concept] OR "cetamolol"[All Fields]) OR (("chlorthalidone"[MeSH Terms] OR "chlorthalidone"[All Fields] OR "chlortalidone"[All Fields]) AND ("tobanum"[Supplementary Concept] OR "tobanum"[All Fields] OR "cloranolol"[All Fields])) OR "cyanoiodopindolol"[All Fields] OR ("cyanopindolol"[Supplementary Concept] OR "cyanopindolol"[All Fields]) OR ("deacetyltrimepranol"[Supplementary Concept] OR "deacetyltrimepranol"[All Fields] OR "deacetylmetipranolol"[All Fields]) OR ("diacetolol"[Supplementary Concept] OR "diacetolol"[All Fields]) OR ("dihydroalprenolol"[MeSH Terms] OR "dihydroalprenolol"[All Fields]) OR ("labetalol"[MeSH Terms] OR "labetalol"[All Fields] OR "dilevalol"[All Fields]) OR ("epanolol"[Supplementary Concept] OR "epanolol"[All Fields]) OR ("esmolol"[Supplementary Concept] OR "esmolol"[All Fields]) OR ("exaprolol"[Supplementary Concept] OR "exaprolol"[All Fields]) OR ("falintolol"[Supplementary Concept] OR "falintolol"[All Fields]) OR ("flestolol"[Supplementary Concept] OR "flestolol"[All Fields]) OR ("flusoxolol"[Supplementary Concept] OR "flusoxolol"[All Fields]) OR "hydroxycarteolol"[All Fields] OR "hydroxymetoprolol"[All Fields] OR ("indenolol"[Supplementary Concept] OR "indenolol"[All Fields]) OR ("iodocyanopindolol"[MeSH Terms] OR "iodocyanopindolol"[All Fields]) OR "iodopindolol"[All Fields] OR ("labetalol"[MeSH Terms] OR "labetalol"[All Fields]) OR ("landiolol"[Supplementary Concept] OR "landiolol"[All Fields]) OR ("levobunolol"[MeSH Terms] OR "levobunolol"[All Fields]) OR ("moprolol"[Supplementary Concept] OR "moprolol"[All Fields] OR "levomoprolol"[All Fields]) OR ("medroxalol"[Supplementary Concept] OR "medroxalol"[All	1.848

	Fields]) OR ("mepindolol"[Supplementary Concept] OR "mepindolol"[All Fields]) OR "methylthiopropranolol"[All Fields] OR ("metipranolol"[MeSH Terms] OR "metipranolol"[All Fields]) OR ("metoprolol"[MeSH Terms] OR "metoprolol"[All Fields]) OR ("moprolol"[Supplementary Concept] OR "moprolol"[All Fields]) OR ("nadolol"[MeSH Terms] OR "nadolol"[All Fields]) OR ("oxprenolol"[MeSH Terms] OR "oxprenolol"[All Fields]) OR ("penbutolol"[MeSH Terms] OR "penbutolol"[All Fields]) OR ("pindolol"[MeSH Terms] OR "pindolol"[All Fields]) OR ("nadolol"[MeSH Terms] OR "nadolol"[All Fields]) OR ("nebivolol"[MeSH Terms] OR "nebivolol"[All Fields]) OR ("nifenalol"[Supplementary Concept] OR "nifenalol"[All Fields]) OR ("nipradilol"[Supplementary Concept] OR "nipradilol"[All Fields]) OR ("oxprenolol"[MeSH Terms] OR "oxprenolol"[All Fields]) OR ("pafenolol"[Supplementary Concept] OR "pafenolol"[All Fields]) OR ("pamatolol"[Supplementary Concept] OR "pamatolol"[All Fields]) OR ("penbutolol"[MeSH Terms] OR "penbutolol"[All Fields]) OR ("pindolol"[MeSH Terms] OR "pindolol"[All Fields]) OR ("practolol"[MeSH Terms] OR "practolol"[All Fields]) OR ("primidolol"[Supplementary Concept] OR "primidolol"[All Fields]) OR ("prizidilol"[Supplementary Concept] OR "prizidilol"[All Fields]) OR ("procinolol"[Supplementary Concept] OR "procinolol"[All Fields]) OR ("pronethalol"[Supplementary Concept] OR "pronethalol"[All Fields] OR "pronetalol"[All Fields]) OR ("propranolol"[MeSH Terms] OR "propranolol"[All Fields] OR "propranolol s"[All Fields] OR "propranolols"[All Fields]) OR ("proxodolol"[Supplementary Concept] OR "proxodolol"[All Fields]) OR ("ridazolol"[Supplementary Concept] OR "ridazolol"[All Fields]) OR ("soquinolol"[Supplementary Concept] OR "soquinolol"[All Fields]) OR ("sotalol"[MeSH Terms] OR "sotalol"[All Fields] OR "sotalol s"[All Fields]) OR ("spirendolol"[Supplementary Concept] OR "spirendolol"[All Fields]) OR ("talinolol"[Supplementary Concept] OR "talinolol"[All Fields]) OR ("tertatolol"[Supplementary Concept] OR "tertatolol"[All Fields]) OR ("tienoxolol"[Supplementary Concept] OR "tienoxolol"[All Fields]) OR ("tilisolol"[Supplementary Concept] OR "tilisolol"[All Fields]) OR ("timolol"[MeSH Terms] OR "timolol"[All Fields]) OR ("tolamolol"[Supplementary Concept] OR "tolamolol"[All Fields]) OR ("toliprolol"[Supplementary Concept] OR "toliprolol"[All Fields]) OR ("xibenolol"[Supplementary Concept] OR "xibenolol"[All Fields])<br>#3 ("beta"[All Fields] AND "adj2"[All Fields]) AND ("adrenergic"[All Fields] OR "antagonist"[All Fields] OR "block"[All Fields] OR "receptor"[All Fields])<br>#4 (("hypertension"[MeSH Terms] OR "white coat hypertension"[MeSH Terms] OR "hypertension"[Title/Abstract] OR "essential hypertension"[MeSH Terms]) AND "acute hypertension"[Title/Abstract]) OR "arterial hypertension"[Title/Abstract] OR "blood pressure high"[Title/Abstract] OR "high blood pressure"[Title/Abstract]<br>(#1 OR #2 OR #3) AND #4	
EMBASE<br>01/08/2024	#1. beta AND adrenergic AND ('receptor'/exp OR receptor) AND blocking AND ('agent'/exp OR agent)<br>#2. ('acebutolol'/exp OR acebutolol OR 'adimolol'/exp OR adimolol OR 'afurolol'/exp OR afurolol OR 'alprenolol'/exp OR alprenolol OR 'amosulalol'/exp OR amosulalol OR 'arotinolol'/exp OR arotinolol OR 'atenolol'/exp OR atenolol OR 'befunolol'/exp OR befunolol OR<br>'betaxolol'/exp OR betaxolol OR 'bevantolol'/exp OR bevantolol OR 'bisoprolol'/exp OR bisoprolol OR 'bopindolol'/exp OR bopindolol OR	4.350

	'bornaprolol'/exp OR bornaprolol OR 'brefonalol'/exp OR brefonalol OR 'bucindolol'/exp OR bucindolol OR 'bucumolol'/exp OR bucumolol OR 'bufetolol'/exp OR bufetolol OR 'bufuralol'/exp OR bufuralol OR 'bunitrolol'/exp OR bunitrolol OR 'bunolol'/exp OR bunolol OR<br>'bupranolol'/exp OR bupranolol OR 'butofilolol'/exp OR butofilolol OR 'butoxamine'/exp OR butoxamine OR 'carazolol'/exp OR carazolol OR 'carteolol'/exp OR carteolol OR 'carvedilol'/exp OR carvedilol OR 'celiprolol'/exp OR celiprolol OR 'cetamolol'/exp OR cetamolol OR 'chlortalidone'/exp OR chlortalidone) AND ('cloranolol'/exp OR cloranolol) OR 'cyanoiodopindolol'/exp OR cyanoiodopindolol OR 'cyanopindolol'/exp OR cyanopindolol OR 'deacetylmetipranolol'/exp OR deacetylmetipranolol OR 'diacetolol'/exp OR diacetolol OR 'dihydroalprenolol'/exp OR dihydroalprenolol OR 'dilevalol'/exp OR dilevalol OR 'epanolol'/exp OR epanolol OR 'esmolol'/exp OR esmolol OR 'exaprolol'/exp OR exaprolol OR 'falintolol'/exp OR falintolol OR 'flestolol'/exp OR flestolol OR 'flusoxolol'/exp OR flusoxolol OR hydroxybenzylpinodolol OR hydroxycarteolol OR hydroxymetoprolol OR 'indenolol'/exp OR indenolol OR 'iodocyanopindolol'/exp OR iodocyanopindolol OR 'iodopindolol'/exp OR iodopindolol OR 'iprocrolol'/exp OR iprocrolol OR 'isoxaprolol'/exp OR isoxaprolol OR 'labetalol'/exp OR labetalol OR 'landiolol'/exp OR landiolol OR 'levobunolol'/exp OR levobunolol OR 'levomoprolol'/exp OR levomoprolol OR 'medroxalol'/exp OR medroxalol OR 'mepindolol'/exp OR mepindolol OR methylthiopropranolol OR 'metipranolol'/exp OR metipranolol OR 'metoprolol'/exp OR metoprolol OR 'moprolol'/exp OR moprolol OR 'nadolol'/exp OR nadolol OR 'nebivolol'/exp OR nebivolol OR 'nifenalol'/exp OR nifenalol OR 'nipradilol'/exp OR nipradilol OR 'oxprenolol'/exp OR oxprenolol OR 'pafenolol'/exp OR pafenolol OR 'pamatolol'/exp OR pamatolol OR 'penbutolol'/exp OR penbutolol OR 'pindolol'/exp OR pindolol OR 'practolol'/exp OR practolol OR 'primidolol'/exp<br>OR primidolol OR 'prizidilol'/exp OR prizidilol OR 'procinolol'/exp OR procinolol OR 'pronetalol'/exp OR pronetalol OR 'propranolol'/exp OR propranolol OR 'proxodolol'/exp OR proxodolol OR 'ridazolol'/exp OR ridazolol OR 'salcardolol'/exp OR salcardolol OR 'soquinolol'/exp OR soquinolol OR 'sotalol'/exp OR sotalol OR 'spirendolol'/exp OR spirendolol OR 'talinolol'/exp OR talinolol OR 'tertatolol'/exp OR tertatolol OR 'tienoxolol'/exp OR tienoxolol OR 'tilisolol'/exp OR tilisolol OR 'timolol'/exp OR timolol OR 'tolamolol'/exp OR tolamolol OR 'toliprolol'/exp OR toliprolol OR 'tribendilol'/exp OR tribendilol OR 'xibenolol'/exp OR xibenolol<br>#3. beta AND adj2 AND (adrenergic? OR antagonist? OR block$ OR receptor?)<br>#4. #1 OR #2 OR #3<br>#5. hypertens$.<br>#6. 'hypertension'/exp<br>#7. 'blood pressure'/exp<br>#8. blood AND pressure AND o AND bloodpressure.mp.<br>#9. #5 OR #6 OR #7 OR #8<br>#10. #4 AND #9<br>#11. 'randomized controlled trial' AND ([controlled clinical trial]/lim OR [randomized controlled trial]/lim) AND [article]/lim AND [humans]/lim AND [embase]/lim	

	#12. 'crossover procedure'<br>#13. 'double blind procedure'<br>#14. randomi?ed OR randomly<br>#15. crossover$ OR cross-over$<br>#16. placebo$.ab.<br>#17. doubl$ AND adj AND blind$<br>#18. assign$.ab.<br>#19. allocat$<br>#20. exp AND animal OR animal.hw. OR nonhuman<br>#21. (exp AND human OR human) AND cell OR human OR humans<br>#22. #11 OR #12 OR #13 OR #14 OR #15 OR #16 OR #17 OR #18 OR #19<br>#23. #20 NOT #21<br>#24. #22 NOT #23<br>#25. #10 AND #24<br>#26. #10 AND #24 AND [2015-2024]/py	
Cochrane library 01/08/2024	#1 (adrenergic beta-antagonist*)<br>#2 (beta-blocker*)<br>#3 (beta adrenergic blocker*)<br>#4 (adrenergic beta receptor blocker*)<br>#5 (beta adrenergic receptor blocker*)<br>#1 OR #2 OR #3 OR #4 OR #5	

Figura S2 – Figura PRISMA da seleção de ensaios clínicos randomizados.

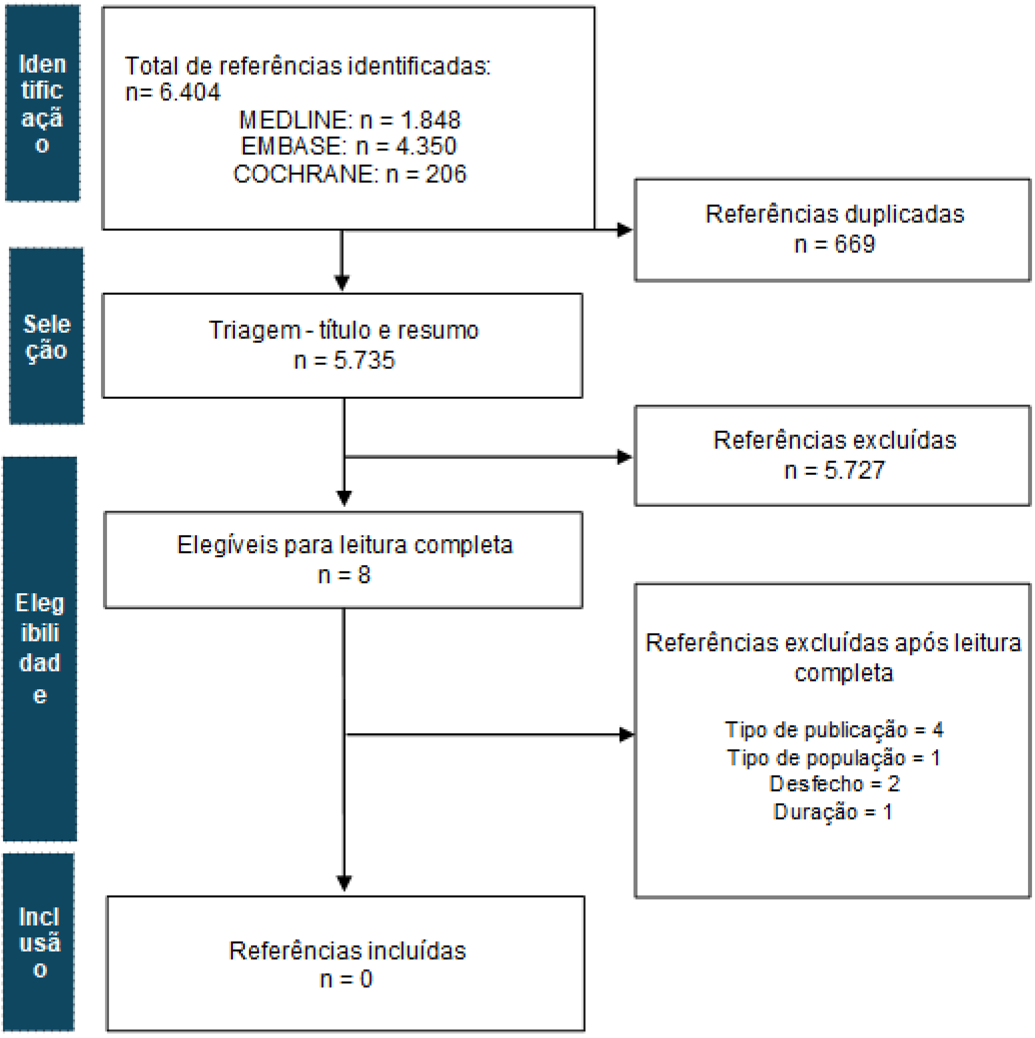

Tabela S3 - revisões sistemáticas excluídas na seleção por texto completo e motivos da exclusão.

Título	Ano	Autores	Site	Motivo exclusão
A meta-analytical comparison of atenolol with angiotensin-converting enzyme inhibitors on arterial stiffness, peripheral blood pressure and heart rate in hypertensive patients.	2017	Xie H and Luo G and Zheng Y and Peng F and Xie L	https://pubmed.ncbi.nlm.nih.gov/28534649/	Desfecho
Blood pressure lowering efficacy of beta-1 selective beta blockers for primary hypertension.	2016	Wong GW and Boyda HN and Wright JM	https://pubmed.ncbi.nlm.nih.gov/26961574/	Desfecho
Blood pressure lowering efficacy of partial agonist beta blocker monotherapy for primary hypertension.	2014	Wong GW and Boyda HN and Wright JM	https://pubmed.ncbi.nlm.nih.gov/25427719/	Desfecho
Comparative effectiveness of fourth-line anti-hypertensive agents in resistant hypertension: A systematic review and meta-analysis.	2017	Sinnott SJ and Tomlinson LA and Root AA and Mathur R and Mansfield KE and Smeeth L and Douglas IJ	https://pubmed.ncbi.nlm.nih.gov/27856806/	População
Effects of nebivolol versus other antihypertensive drugs on the endothelial dysfunction in patients with essential hypertension.	2020	Li B and Zhang Q and Zhang H and Wang C and Xiu R	https://pubmed.ncbi.nlm.nih.gov/32342981/	Desfecho
Carvedilol and bisoprolol as initial therapy for adult hypertension without compelling indications.	2019	Kishi T and Fujii E	https://pubmed.ncbi.nlm.nih.gov/30948819/	Desfecho
Clinical pharmacokinetics of nadolol: A systematic review.	2022	Kalsoom S and Zamir A and Rehman AU and Ashraf W and Imran I and Saeed H	https://pubmed.ncbi.nlm.nih.gov/36040016/	Desfecho

		and Majeed A and Alqahtani F and Rasool MF			
COMPARATIVE EFFICACY AND ACCEPTABILITY OF DIFFERENT ANTIHYPERTENSIVE DRUG CLASSES FOR CARDIOVASCULAR DISEASE PREVENTION: A SYSTEMATIC REVIEW AND NETWORK META-ANALYSIS	2023	BrunstrÃ¶m, M. and BrunstrÃ¶m, A. and Jussil, H. and Panagiotopoulou, K. and Chaimani, A. and Carlberg, B.	https://www.embase.com/search/results?subaction=viewrecord&id=L641880968&from=export	U2 - L641880968	Tipo de estudo
Application of Î²-blockers for hypertension	2022	Cui, S. and Cui, Z.	https://www.embase.com/search/results?subaction=viewrecord&id=L2018248350&from=export	U2 - L2018248350	Idioma e artigo não localizado
COMPREHENSIVE COMPARATIVE EFFECTIVENESS AND SAFETY OF FIRST-LINE ANTIHYPERTENSIVE MONOTHERAPY IN HYPERTENSIVE PATIENTS: A LARGE-SCALE MULTINATIONAL PARTICIPANT-LEVEL ASSESSMENT	2022	Lu, Y. and Khera, R. and Liu, Y. and Chen, R. and Schuemie, M. and Hripcsak, G. and Suchard, M. and Krumholz, H.M.	https://www.embase.com/search/results?subaction=viewrecord&id=L2017302715&from=export	U2 - L2017302715	Tipo de estudo
First-line drug classes for hypertension in adults: a network meta-analysis	2020	Lunny, C. and Heran, B.S. and Beaumier, J. and Salzwedel, D.M. and Adams, S.P. and Jauca, C.D. and Musini, V.M. and Wright, J.M.	https://www.embase.com/search/results?subaction=viewrecord&id=L632931003&from=export	U2 - L632931003	Tipo de estudo
Legacy effect on mortality in antihypertensive drug comparison trials: A meta-analysis	2019	Thomopoulos, C. and Antza, C.	https://www.embase.com/search/results?subaction=viewrecord&id=L629057987&from=export	U2 - L629057987	Tipo de estudo
Blood pressure lowering for prevention of cardiovascular disease and death: A systematic review and meta-analysis	2016	Ettehad, D. and Emdin, C. and Kiran, A. and Anderson, S. and Callender, T. and	https://www.embase.com/search/results?subaction=viewrecord&id=L612284812&from=export	U2 - L612284812	Tipo de estudo

		Emberson, J. and Chalmers, J. and Rodgers, A. and Rahimi, K.			
Beta-blockers as first-line therapy for hypertension?	2013	Scholze, J.E.	https://www.embase.com/search/results?subaction=viewrecord&id=L52466392&from=export	U2 - L52466392	Idioma
Carvedilol versus metoprolol for primary hypertension: A systematic review	2013	Niu, X.-W. and Xu, H. and He, S.-L. and Chen, D. and Yan, D. and He, Z.-Y. and Yao, Y.-L.	https://www.embase.com/search/results?subaction=viewrecord&id=L373923706&from=export	U2 - L373923706	Desfecho

Tabela S4 - Ensaios clínicos excluídos na etapa de leitura do texto completo e motivos da exclusão.

Título	Ano	Autores	Site	Motivo exclusão	doi
Combination of carvedilol with variceal band ligation in prevention of first variceal bleed in Child-Turcotte-Pugh B and C cirrhosis with high-risk oesophageal varices: the 'CAVARLY TRIAL'.	2024	Tevethia HV and Pande A and Vijayaraghavan R and Kumar G and Sarin SK	https://pubmed.ncbi.nlm.nih.gov/39067870/	População	10.1136/gutjnl-2023-331181
Systematic Review Article: New Drug Strategies for Treating Resistant Hypertension-the Importance of a Mechanistic, Personalized Approach.	2024	Nardoianni G and Pala B and Scoccia A and Volpe M and Barbato E and Tocci G	https://pubmed.ncbi.nlm.nih.gov/38616212/	Tipo de estudo	10.1007/s40292-024-00634-4
Effectiveness and safety assessment of beta-blockers, calcium channel blockers, and angiotensin receptor blockers in	2021	Solanki N and Pandit D and Desai S	https://pubmed.ncbi.nlm.nih.gov/34849291/	Duração do estudo	

hypertensive patients: a prospective study.					
Long-term mortality after blood pressure-lowering and lipid-lowering treatment in patients with hypertension in the Anglo-Scandinavian Cardiac Outcomes Trial (ASCOT) Legacy study: 16-year follow-up results of a randomised factorial trial.	2018	Gupta A and Mackay J and Whitehouse A and Godec T and Collier T and Pocock S and Poulter N and Sever P	https://pubmed.ncbi.nlm.nih.gov/30158072/	Tipo de estudo	10.1016/S0140-6736(18)31776-8
A meta-analytical comparison of atenolol with angiotensin-converting enzyme inhibitors on arterial stiffness, peripheral blood pressure and heart rate in hypertensive patients.	2017	Xie H and Luo G and Zheng Y and Peng F and Xie L	https://pubmed.ncbi.nlm.nih.gov/28534649/	Desfecho	10.1080/10641963.2016.1267188
Beta-Adrenergic Receptor Blockers in Hypertension: Alive and Well.	2016	Frishman WH	https://pubmed.ncbi.nlm.nih.gov/27984052/	Artigo de revisão	10.1016/j.pcad.2016.10.005
Pharmacogenomic Genome-Wide Meta-Analysis of Blood Pressure Response to Î²-Blockers in Hypertensive African Americans.	2016	Gong Y and Wang Z and Beitelshees AL and McDonough CW and Langaee TY and Hall K and Schmidt SO and Curry RW Jr and Gums JG and Bailey KR and Boerwinkle E and Chapman AB and Turner ST and Cooper-DeHoff RM and Johnson JA	https://pubmed.ncbi.nlm.nih.gov/26729753/	Desfecho	10.1161/HYPERTENSIONAHA.115.06345

Efficacy and Safety of Antihypertensive Drug Classes: the Systolic Blood Pressure Intervention Trial (SPRINT)	2020	Byrne, C and Varshney, A and Almarzooq, Z and Kragholm, KH and Krogager, ML and Vaduganathan, M and Biering-Srensen, T and Olsen, MH and Bhatt, DL and Pareek, M	https://www.cochranelibrary.com/central/doi/10.1002/central/CN-02259489/full	Tipo de estudo	10.1161/circ.142.suppl_3.14373

Tabela S5 - PRISMA da revisão sistemática.

**Item**	**Descrição**	Local
TÍTULO - 1	Identificar o relatório como uma revisão sistemática.	1
RESUMO - 2	Ver o checklist PRISMA 2020 para resumos.	2
INTRODUÇÃO - Racional - 3	Descrever a justificativa para a revisão no contexto do conhecimento existente.	3
INTRODUÇÃO - Objetivos - 4	Fornecer uma declaração explícita dos objetivos ou perguntas que a revisão aborda.	4
MÉTODOS - Critérios de Elegibilidade - 5	Especificar os critérios de inclusão e exclusão da revisão e como os estudos foram agrupados.	5
MÉTODOS - Fontes de Informação - 6	Especificar todas as bases de dados, registros, sites, organizações e outras fontes consultadas.	5
MÉTODOS - Estratégia de Busca - 7	Apresentar as estratégias de busca completas para todas as bases de dados e sites.	S1, S2
MÉTODOS - Processo de Seleção - 8	Especificar os métodos para decidir se um estudo atendia aos critérios de inclusão da revisão.	5
MÉTODOS - Processo de Coleta de Dados - 9	Especificar os métodos para coleta de dados dos relatórios, incluindo detalhes de ferramentas.	6, Material Suplementar
MÉTODOS - Itens de Dados - 10a	Listar e definir todos os desfechos para os quais os dados foram procurados.	5

MÉTODOS - Outras Variáveis de Dados - 10b	Listar e definir todas as outras variáveis para as quais os dados foram procurados (ex: características dos participantes, fontes de financiamento).	Tabelas 1 e 2
MÉTODOS - Avaliação de Risco de Viés - 11	Especificar os métodos para avaliar o risco de viés nos estudos incluídos.	6, Material Suplementar
MÉTODOS - Medidas de Efeito - 12	Especificar para cada desfecho as medidas de efeito usadas.	7
MÉTODOS - Métodos de Síntese - 13a	Descrever os processos usados para decidir quais estudos eram elegíveis para cada síntese.	5
MÉTODOS - Preparação de Dados - 13b	Descrever métodos necessários para preparar os dados para apresentação ou síntese.	Material Suplementar
MÉTODOS - Exibição de Resultados - 13c	Descrever métodos para exibir visualmente os resultados dos estudos individuais.	Material Suplementar
MÉTODOS - Métodos de Síntese - 13d	Descrever métodos usados para sintetizar resultados e fornecer justificativa para as escolhas.	6, Material Suplementar
MÉTODOS - Heterogeneidade - 13e	Descrever métodos para explorar causas de heterogeneidade entre resultados dos estudos.	Material Suplementar
MÉTODOS - Análises de Sensibilidade - 13f	Descrever análises de sensibilidade conduzidas para avaliar a robustez dos resultados.	8
MÉTODOS - Avaliação de Viés de Relato - 14	Descrever métodos usados para avaliar o risco de viés devido a resultados ausentes.	6, Material Suplementar
MÉTODOS - Avaliação de Certeza - 15	Descrever métodos usados para avaliar a certeza (ou confiança) no corpo de evidências.	6, Material Suplementar

RESULTADOS - Seleção de Estudos - 16a	Descrever os resultados do processo de busca e seleção dos estudos.	7, Material Suplementar
RESULTADOS - Estudos Excluídos - 16b	Citar estudos que atendiam aos critérios, mas foram excluídos, e justificar exclusões.	7, Material Suplementar
RESULTADOS - Características dos Estudos - 17	Citar cada estudo incluído e apresentar suas características.	Tabelas 1 e 2
RESULTADOS - Risco de Viés nos Estudos - 18	Apresentar as avaliações de risco de viés para cada estudo incluído.	Figura 6
RESULTADOS - Resultados dos Estudos Individuais - 19	Apresentar, para cada estudo, as estatísticas resumidas e estimativas de efeito.	Figuras 1 a 5
RESULTADOS - Sínteses de Resultados - 20a	Resumir características e risco de viés entre estudos incluídos em cada síntese.	Figura 6
RESULTADOS - Estatísticas de Síntese - 20b	Apresentar resultados de todas as sínteses estatísticas conduzidas.	Figuras 1 a 5 e 8
RESULTADOS - Heterogeneidade - 20c	Apresentar resultados de investigações de causas de heterogeneidade.	NA
RESULTADOS - Análises de Sensibilidade - 20d	Apresentar resultados de análises de sensibilidade conduzidas para avaliar robustez dos resultados.	Figura 8

RESULTADOS - Viés de Relato - 21	Apresentar avaliações de risco de viés devido a resultados ausentes para cada síntese.	NA
RESULTADOS - Certeza das Evidências - 22	Apresentar avaliações de certeza no corpo de evidências para cada desfecho avaliado.	Figura 7
DISCUSSÃO - Interpretação dos Resultados - 23a	Interpretar os resultados no contexto de outras evidências.	10 a 13
DISCUSSÃO - Limitações da Evidência - 23b	Discutir quaisquer limitações da evidência incluída na revisão.	14
DISCUSSÃO - Limitações da Revisão - 23c	Discutir quaisquer limitações dos processos de revisão usados.	14
DISCUSSÃO - Implicações - 23d	Discutir implicações dos resultados para prática, política e pesquisa futura.	1, 14
OUTRAS INFORMAÇÕES - Registro e Protocolo - 24a	Fornecer informações de registro da revisão, incluindo nome e número de registro.	4
OUTRAS INFORMAÇÕES - Acesso ao Protocolo - 24b	Indicar onde o protocolo da revisão pode ser acessado.	4

OUTRAS INFORMAÇÕES - Emendas ao Protocolo - 24c	Descrever e explicar quaisquer emendas às informações fornecidas no protocolo.	NA
OUTRAS INFORMAÇÕES - Suporte - 25	Descrever fontes de apoio financeiro ou não-financeiro para a revisão.	6
OUTRAS INFORMAÇÕES - Conflitos de Interesse - 26	Declarar quaisquer conflitos de interesse dos autores da revisão.	6
OUTRAS INFORMAÇÕES - Disponibilidade de Dados e Materiais - 27	Indicar quais materiais (dados, códigos) estão publicamente disponíveis e onde encontrá-los.	NA

Abreviatura: NA – Não aplicável.
