# Supplementary material for: Revisão Sistemática Sobre a Eficácia do Atenolol no Tratamento Anti-Hipertensivo: Recomendação da Sociedade Brasileira de Cardiologia
Source: Arq Bras Cardiol. 2025 Sep 8;122(9):e20250034. [Article in Portuguese] doi: 10.36660/abc.20250034 (PMC12677729; doi:10.36660/abc.20250034)
Supplement: Translation

# Translation: Systematic Review on the Efficacy of Atenolol in Antihypertensive Treatment: Recommendation from the Brazilian Society of Cardiology

**DOI:** 10.36660/abc.20250034i

**Authors:** Andréa Araujo Brandão, Cibele Isaac Saad Rodrigues, Wilson Nadruz, Paulo Cesar B Veiga Jardim, Fernando Nobre, Sergio Emanuel Kaiser, Otavio Rizzi Coelho, Fernanda Marciano Consolim Colombo, Leonardo Castro Luna, Andressa Braga, Quenia Dias Morais, Luiz Bortolotto

**Affiliations:** 1 Brasil Universidade do Estado do Rio de Janeiro, Rio de Janeiro, RJ – Brasil; 2 Brasil Pontifícia Universidade Católica de São Paulo, Sorocaba, SP – Brasil; 3 Brasil Universidade Estadual de Campinas, Campinas, SP – Brasil; 4 Brasil Universidade Federal de Goiás, Goiânia, GO – Brasil; 5 Brasil Universidade de São Paulo (USP), Ribeirão Preto, SP – Brasil; 6 Brasil Universidade do Estado do Rio de Janeiro – Clinex, Rio de Janeiro, RJ – Brasil; 7 Brasil Universidade Estadual de Campinas (UNICAMP), Campinas, SP – Brasil; 8 Brasil Instituto do Coração do Hospital das Clínicas da Faculdade de Medicina da Universidade de São Paulo, São Paulo, SP – Brasil; 9 Brasil Instituto Nacional de Cardiologia, Rio de Janeiro, RJ – Brasil

**Keywords:** Hypertension, Systematic Review, Meta-Analysis, Drug Therapy, Atenolol

## Abstract

**Background:**

Hypertension (HTN) is a global public health issue, with high prevalence and a significant impact on cardiovascular morbidity and mortality. Cardioselective beta-blockers, such as atenolol, are widely used in the treatment of HTN, although their indication as first-line therapy remains controversial.

**Objective:**

To evaluate the efficacy and safety of atenolol in the treatment of primary HTN, compared with other first-line classes of antihypertensive drugs.

**Methods:**

A systematic review was conducted based on a research question structured using the PICO format. Randomized clinical trials comparing atenolol with other antihypertensive agents were included. Searches were performed in three international databases. Methodological quality was assessed using the RoB 2 tool, and the certainty of evidence was rated using the GRADE system. The primary composite outcome was the occurrence of major cardiovascular events. Secondary outcomes included all-cause mortality, acute myocardial infarction, and stroke, each analyzed separately.

**Results:**

Seven clinical trials met the inclusion criteria. Compared with amlodipine and losartan, atenolol was associated with a slightly higher incidence of cardiovascular events, with low and moderate certainty of evidence, respectively. The combination of hydrochlorothiazide and amiloride demonstrated a greater reduction in cardiovascular events compared to atenolol, although with very low certainty of evidence. Blood pressure (BP) reduction was similar across the compared treatments.

**Conclusions:**

Despite the limitations of available evidence, atenolol showed comparable efficacy in BP reduction, with small differences in cardiovascular outcomes favoring other antihypertensive classes. Its use may be considered among the options for combination therapy in the treatment of primary HTN in adults. Other beta-blockers were not evaluated in this systematic review.

## Introduction

Hypertension (HTN) is one of the leading global and national public health concerns, responsible for approximately 10 million deaths per year worldwide, primarily due to cardiovascular complications such as acute myocardial infarction (AMI) and stroke.^1^ In Brazil, the reported prevalence of HTN was 21.4% (95% CI: 20.8 to 22.0) based on self-report; 22.8% (95% CI: 22.1 to 23.4) based on measured blood pressure (BP); and 32.3% (95% CI: 31.7 to 33.0) when considering either measured BP and/or reported use of antihypertensive medication(s).^2^ Effective strategies for prevention, early diagnosis, and BP control are essential to mitigate the impact of HTN, especially among vulnerable populations.^3^

Pharmacological treatment of HTN remains a major challenge. Prominent studies on the topic, such as ACCORD^4^ and SPRINT,^5^ which established more intensive BP control targets in the intervention groups (systolic BP <120 mmHg), have shown that on average individuals with HTN require approximately three combined medications to reach that goal. In addition to increasing treatment costs and affecting patient adherence, this also highlights the need for a broader range of therapeutic options with proven clinical efficacy.

Beta-blockers (BBs) are a heterogeneous group of drugs that act by blocking peripheral and central beta-adrenergic receptors as well as by inhibiting renin release from the juxtaglomerular apparatus in the kidney.^6,7^ However, they differ in terms of receptor selectivity and additional pharmacological effects. Introduced for clinical use in the 1960s, they became the most widely prescribed class of antihypertensive drugs during the 1980s.^8,9^ Later, in the 2000s, primary studies and meta-analyses were published suggesting that BBs, when analyzed as a group, were less effective than other antihypertensive classes — particularly in cerebrovascular protection — thereby relegating BBs to a secondary role in HTN management.^10,11^ A current point of concern is that these studies assumed equivalent clinical effects among different BBs in HTN, and at the time, the reviews did not account for the quality of the included studies (i.e., the certainty of evidence) in their conclusions.

With the aim of addressing this gap in the treatment of HTN, and considering the best available scientific evidence, the Brazilian Society of Cardiology (*Sociedade Brasileira de Cardiologia*, SBC) commissioned the development of a Clinical Recommendation on the use of atenolol — the most widely used and available medication within the BB class — for the treatment of individuals with HTN.

## Methods

To support the SBC Clinical Recommendation, a systematic review was conducted. The research question, structured in the PICO format, was: what is the efficacy and safety of treating HTN with atenolol compared to other antihypertensive agents? The study protocol was registered in the International Prospective Register of Systematic Reviews (PROSPERO) under the number CRD42024563608.

The rapid systematic review, the methodology employed in this document, belongs to the broader family of systematic reviews but differs from traditional approaches. This methodology was developed to maintain appropriate methodological rigor in the search for the best available evidence, while incorporating adaptations that allow for a shorter execution time. The main differences include restricting the language of primary articles to English; searching the grey literature only through references of retrieved studies and expert consultation; and using artificial intelligence to facilitate study selection and data extraction. Overall, this type of review is useful for supporting medical societies or health care institutions in making decisions that are sensitive, transparent, systematic, and evidence-based, guided by a question structured in the PICO format. Leading institutions in the field of methodology have described the standards for conducting this type of systematic review.^12-14^

The selection of atenolol for this systematic review, instead of other BBs, was a consensus among the authors and was based on objective criteria, such as: being widely known and available throughout the country; being distributed free of charge through the basic component of the Brazilian Unified Health System (*Sistema Único de Saúde*, SUS); and, consequently, being included in the National List of Essential Medicines (ReNaMe 2024) and the Popular Pharmacy Program of Brazil (*Programa Farmácia Popular do Brasil*), coordinated by the Brazilian Ministry of Health.^15,16^ Additionally, atenolol is the BB with the largest number of studies in HTN, showing a significant difference compared to others, such as metoprolol or propranolol, which have few comparative studies in patients with HTN.^11^ The pharmacological effects of sympathetic blockade vary across the different molecules in this class,^9^ and may not be homogeneous in terms of BP reduction and clinical outcomes, which is why they were not grouped together in a single analysis.

The inclusion criteria were: (1) systematic reviews of randomized clinical trials (RCTs), or original RCTs, with at least two comparative arms; (2) presence of atenolol as the main drug in one of the intervention groups; (3) reporting of data on at least one of the outcomes of interest; (4) inclusion of adult patients aged 18 years or older; and (5) a minimum follow-up duration of 1 year.

The exclusion criteria were: (1) RCTs in which atenolol was used as a second-line medication; (2) other clinical study designs; (3) RCTs with a pre-specified crossover design; and (4) post hoc analyses of RCTs that did not present new or additional data relevant to the outcomes analyzed.

The primary outcome was a composite of major cardiovascular events, including all-cause mortality, stroke, and AMI. The selected secondary outcomes were the individual components of the primary outcome, in addition to adverse events potentially reported in the original studies.

Searches for studies related to the PICO question were conducted in three databases: PubMed, Embase, and the Cochrane Library.

Selection, data extraction of key study characteristics, and quality assessment were conducted independently by two experienced researchers. Risk of bias was also assessed independently using the Risk of Bias 2 (RoB 2) tool.^17^ In cases of disagreement, a third methodologist was consulted. The certainty of the evidence and the strength of the recommendations were determined based on the Grading of Recommendations, Assessment, Development and Evaluations (GRADE) framework.^18^

The summary of effect for the comparisons between atenolol and other drugs was estimated through a network meta-analysis.

A network meta-analysis is an advanced statistical technique that combines both direct and indirect evidence from multiple comparative studies to assess the efficacy of different interventions simultaneously — even when not all have been directly compared with each other.^19,20^ However, this review included only direct comparisons between atenolol and six alternative treatments, in addition to a no-treatment arm. No indirect comparisons (e.g., comparing drugs A and C via studies that compared A with B and B with C) or full network comparisons were performed. Meta-analysis was conducted using the MetaInsight software.^21^

A detailed description of the methodology used in this rapid systematic review is available in the supplementary material, along with the Preferred Reporting Items for Systematic Reviews and Meta-Analyses (PRISMA) checklist.

A 5% threshold was adopted as the minimally important difference (MID) to define clinical relevance. Therefore, if a treatment demonstrates an effect whose confidence interval does not cross the line of no effect, it is considered statistically significant. However, to be deemed clinically relevant, the 95% CI must not touch the MID threshold of 5%.^22,23^ This threshold was established in advance by the Recommendation Panel, prior to the presentation and awareness of the results.

The clinical recommendation was established by consensus during a meeting of a Recommendation Panel composed of experts appointed by the sponsoring organization. The entire project was overseen and funded by SBC, while the systematic review and the clinical recommendation process were conducted by an independent team of methodologists (LL, AB, and QD). The team of methodologists and the authors declare that they have no relevant conflicts of interest related to this review.

## Results

The initial search for systematic reviews identified 815 publications. After removing duplicates, screening titles and abstracts, and applying the eligibility criteria, 17 articles were selected for full-text review. Of these, none specifically addressed the proposed research question; however, two documents had potential as sources of references for studies comparing atenolol with other classes of antihypertensive drugs in the treatment of HTN^24,25^ (Figure S1 – PRISMA flowchart of systematic review selection).

Among these two studies, the review by Wiysonge et al.^24^ was selected as the main reference for identifying primary studies and for guiding the search strategy, as it was the most recent and comprehensive publication. This review included seven primary studies comparing atenolol with other classes of antihypertensive drugs, which were eligible for inclusion in the new review. Furthermore, its search strategy served as a model for updating the search to include RCTs published after 2017.^24^

The second database search, now focused on randomized controlled trials (RCTs) to update the selection from the reference systematic review, identified 6,404 records. Of these, 669 were duplicates, and 5,727 studies were excluded based on titles and abstracts. In addition, a manual search of the reference lists from the systematic reviews selected in the first search was conducted. In this second phase, eight studies were selected for full-text review. However, no additional RCTs beyond the seven primary studies included in Wiysonge et al.^24^ met the inclusion criteria based on the PICO question and were therefore not added to the network meta-analysis (Figure S2 – PRISMA flowchart of RCT selection).

The details of the search strategies, the documents excluded after full-text review, and the PRISMA flowcharts are provided in the supplementary material (Tables S1 to S4, Figures S1 and S2).

The updated search did not yield any additional studies evaluating the specific intervention (atenolol). The main characteristics of the seven primary randomized controlled trials included (originally selected in the review by Wiysonge et al.^24^) are summarized in Tables 1 and 2.

The included studies evaluated populations with HTN and varying clinical profiles, with sample sizes ranging from 884 to 22,576 participants and mean ages between 56 and 79 years. The populations comprised individuals with left ventricular hypertrophy, type 2 diabetes mellitus, and additional cardiovascular risk factors, in addition to HTN. Further details of the methodology and study search/selection process can be found in the supplementary material.

Effect summaries are presented in forest plots, comparing different treatments to atenolol (used as the reference). Odds ratios (ORs) and 95% CIs are reported. OR values greater than 1 indicate a higher likelihood of cardiovascular events with atenolol compared to the comparator treatment, whereas OR values less than 1 indicate a lower likelihood of events with atenolol.

Regarding the primary outcome of major cardiovascular events, the combination of amiloride and hydrochlorothiazide (HCTZ) demonstrated a protective profile, with a lower number of cardiovascular events compared to atenolol, exceeding the 5% threshold for the MID. Amlodipine and losartan also showed fewer events compared to atenolol; however, the lower bound of the 95% CI reached the 5% threshold, which reduces confidence in the clinical relevance of these findings (Figure 1).

**Figure 1:** – Composite outcomes: major cardiovascular events (death, stroke, and acute myocardial infarction). Thin red horizontal lines highlight statistically significant differences, while thick red lines indicate clinically relevant differences. The vertical blue lines represent the 5% MID threshold. The certainty of the evidence for each comparison is shown on the right.

For the secondary outcomes — mortality (Figure 2), stroke (Figure 3), and AMI (Figure 4) — the 95% CIs exceeded the MID threshold, indicating results within the range of no clinical relevance. In nearly all cases, the intervals also crossed the line of no effect, suggesting no statistically significant difference between treatment strategies.

**Figure 2:** – Outcome: all-cause mortality according to different antihypertensive treatments compared to atenolol.

**Figure 3:** – Outcome: stroke, according to different antihypertensive treatments compared to atenolol.

**Figure 4:** – Outcome: acute myocardial infarction, according to different antihypertensive treatments compared to atenolol.

Only three of the seven studies reported adverse events (Figure 5). Among them, the comparison with amlodipine was the only one to show a statistically significant difference, with bradycardia occurring more frequently in the atenolol group (OR 16.67; 95% CI: 11.77 to 23.61) — a clinically expected outcome for BBs.

**Figure 5:** – Outcome: adverse events, according to different antihypertensive treatments compared to atenolol.

Risk of bias and certainty of evidence were assessed for the primary composite outcome and for the individual secondary outcomes of all-cause mortality, AMI, and stroke across the seven RCTs. The assessment of risk of bias covered five domains per study: randomization, deviations from intended interventions, missing outcome data, outcome measurement, and selective reporting. Most of the downgrades in risk of bias were due to deviations from intended interventions (D2) and missing data (D3), as seen in the MRCOA26^26^ and COOPE^27^ studies, both classified as high risk of bias. The ASCOT-BPLA28^28^ and UKPDS39^29^ studies showed moderate risk of bias due to issues with blinding and unexplained deviations. The INVEST30,^30^ LIFE,^31^ and ELSA^32^ studies were classified as low risk of bias (Figure 6 and Supplementary Material).

**Figure 6:** – Risk of bias (RoB 2) of the primary studies included in this systematic review. The five evaluated domains are shown, using the following color coding: green for low risk of bias, yellow for moderate risk, and red for high risk of bias. The final judgment per study is also presented, with respective symbols indicating the specific issue that led to a downgrade. Unexplained deviations from treatment (*); Blinding issues, multiple comparators (†); Treatment deviations, loss to follow-up, and lack of protocol (‡); Treatment deviations, missing data not addressed (§). D1: Randomization; D2: Treatment Deviations; D3: Missing Data; D4: Outcome Measurement; D5 Reported Data.

The certainty of the evidence, assessed using the GRADE system, ranged from very low to moderate. For studies comparing atenolol with verapamil, losartan, or lacidipine, the certainty of the evidence was rated as moderate. In contrast, studies comparing atenolol with hydrochlorothiazide plus amiloride or with no treatment were classified as having very low certainty (Figure 7).

**Figure 7:** – GRADE assessment of the primary studies included in this systematic review. HCTZ: hydrochlorothiazide

As a sensitivity analysis, in comparison to the network meta-analysis methodology, a conventional meta-analysis forest plot was generated, comparing atenolol with different antihypertensive classes grouped as a single comparator. Notably, the effect size estimates for each comparison (atenolol vs. other antihypertensives) differed numerically between the network meta-analysis and the conventional meta-analysis. In the latter, where comparator classes were grouped together, the results showed high heterogeneity (Figure 8), reinforcing the argument that antihypertensive classes with distinct mechanisms of action should not be pooled into a single comparator group within a meta-analysis.

**Figure 8:** – Conventional meta-analysis comparing the composite outcome of cardiovascular events between atenolol and other antihypertensive drugs grouped as comparators.

For improved interpretation of the forest plots below, which represent the effects of each treatment strategy, the term “standard” was used to describe the comparator arm in the COOPE study (1998), in which atenolol (with the possible addition of bendroflumethiazide and methyldopa, if needed) was compared to no treatment. In that study, the comparator group would only receive antihypertensive therapy if the patient maintained BP values above 280/120 mmHg or experienced a stroke. The strategy labeled “placebo” refers to the third arm of the MRCOA study (1992), in which patients were allocated to receive atenolol, hydrochlorothiazide plus amiloride, or placebo.

## Discussion

BBs are a class of medications widely used in the treatment of cardiovascular diseases such as HTN, heart failure, coronary artery disease, and arrhythmias. They are also indicated for other conditions, including migraine prophylaxis, essential tremor, and the management of anxiety symptoms.^24,33,34^ These drugs act by blocking beta-adrenergic receptors, thereby reducing the effects of catecholamines on the heart and vascular system.^6^ They also act on the central nervous system and inhibit renin secretion by the juxtaglomerular apparatus in the kidneys.^3^ There are different subclasses of BBs, including selective β_1_ receptor blockers, which have a lower impact on β_2_ receptors (e.g., atenolol, metoprolol, and bisoprolol), and non-selective BBs, which block both β_1_ and β_2_ receptors (e.g., propranolol). In addition, some BBs possess additional properties, such as vasodilatory activity, as observed in carvedilol and nebivolol.^35^ Common side effects of BBs include bradycardia, fatigue, hypotension, bronchospasm (particularly with non-selective agents), and sexual dysfunction.^24^

In the 1970s and 1980s, these medications were considered first-line treatment for HTN, being recommended in 1983 by the World Health Organization in collaboration with the International Society of Hypertension, and in 1988 by the Joint National Committee.^7,8^ However, especially from the 1990s onward, original studies and meta-analyses were published comparing BBs to other classes of antihypertensive drugs, questioning the role of BBs in the treatment of HTN.

In 2005, a systematic review was published that included 13 RCTs with 105,951 participants comparing BBs, as a group, to other classes of antihypertensive drugs. That same review also included seven trials with 27,433 individuals comparing BBs to placebo.^10^ The conclusion categorically stated that the effectiveness of BBs was inferior, with an increased risk of stroke, and that they should therefore no longer be considered first-line agents for the treatment of primary HTN. In this review, the included primary studies assessed different BBs, which were pooled and compared to pooled classes of other antihypertensive drugs, as well as placebo. In some trials, a single study arm included more than one BB. In the final summary of the meta-analysis, no statistically significant difference was found between groups for all-cause mortality or AMI, but a statistically significant difference was observed for stroke — although not clinically relevant, as the 95% confidence interval exceeded the predefined MID threshold of 5%. A secondary analysis specifically comparing atenolol to pooled antihypertensive drug classes showed a statistically significant difference in mortality and a clinically relevant difference in stroke, both favoring the other drug classes. No assessment of the quality of the studies included was performed.

In a systematic review published in 2007, Dahlöf et al.^36^ conducted a meta-analysis of studies evaluating atenolol and other BBs vs. placebo or no treatment. At that time, BBs were no longer recommended as first-line options for HTN. Regarding stroke, results showed a statistically significant protective effect in favor of atenolol, with no significant difference observed for the composite outcome of cardiovascular events. In the discussion, the authors emphasized that the use of BBs reduces cardiovascular risk in HTN when compared to placebo or no treatment. However, they highlighted a clear superiority of losartan and amlodipine in more recent studies at the time — LIFE and ASCOT, respectively — for the outcome of stroke, concluding that BBs were no longer appropriate comparators in new RCTs.

At that time, it was not standard practice to assess the quality of the primary studies; conclusions were based solely on the numerical estimates of benefits and harms associated with each strategy. A second methodological limitation of those studies lies in the comparison of BBs, as a single group, with a pooled group of distinct classes of antihypertensive drugs. This approach is questionable, as calcium channel blockers, thiazide and thiazide-like diuretics, and renin–angiotensin–aldosterone system (RAAS) blockers have different mechanisms of action, side effect profiles, and potentially distinct efficacy outcomes.

In 2017, the Cochrane Collaboration published a systematic review on BBs,^24^ also analyzed as a pooled group, but this time compared with placebo and with other classes of antihypertensive drugs evaluated separately: diuretics, calcium channel blockers, RAAS inhibitors, and alpha-blockers. This review applied the GRADE tool to assess the certainty of evidence for each comparison, incorporating study quality into the final interpretation of results. The main findings showed a statistically significant reduction in stroke and in the composite outcome of cardiovascular events in favor of BBs compared with placebo. However, the 95% CIs exceeded the prespecified 5% threshold for minimal clinically important difference. As a result, this evidence was graded as low certainty. Moreover, when comparing pooled BBs with calcium channel blockers (for stroke and cardiovascular events) and with RAAS inhibitors (for stroke), the alternative treatments showed clinically relevant reductions in events, with certainty of evidence rated as moderate. These results reinforce the ongoing debate over whether the effects of BBs on BP and clinical outcomes, when analyzed as a group, are truly homogeneous — representing a class effect — or whether there are distinct actions among different generations of BBs, which would make pooling them in a single analysis methodologically inappropriate.

In 2020, Thomopoulos et al.^11^ conducted a systematic review on the use of BBs for the treatment of HTN. Although 87 studies were included, only 16 specifically focused on HTN. Of these, seven evaluated atenolol as the active treatment, four investigated propranolol, two analyzed metoprolol, one tested oxprenolol, one grouped atenolol and metoprolol in the same intervention arm, and one assessed any BB. In this review, the vast majority of the included RCTs addressed other conditions, mainly coronary artery disease and heart failure. The authors concluded that “compared with other antihypertensive drugs, BBs appear to be substantially less effective in protecting against stroke and all-cause mortality.” The comparison between pooled BBs and pooled antihypertensive drug classes showed a clinically relevant difference in stroke outcomes favoring the other medications in HTN-specific studies (RR = 1.21; 95% CI: 1.07 to 1.38), and a clinically irrelevant difference for all-cause mortality (RR = 1.06; 95% CI: 1.01 to 1.12). No separate analysis was conducted for atenolol, and heterogeneity was not reported alongside the forest plots. Risk of bias was assessed using the RoB 2 tool; however, the other GRADE components — heterogeneity, indirectness, imprecision, and publication bias — were not considered collectively to determine the overall quality of the evidence (certainty of evidence).

In a 2023 Cochrane Collaboration systematic review^37^ comparing diuretics with other classes of antihypertensive drugs, no differences were observed in the outcomes assessed when compared with BBs — including cardiovascular events, all-cause mortality, stroke, myocardial infarction, and heart failure. The certainty of evidence ranged from low to moderate.

Despite these results, which showed mixed conclusions — some neutral and others unfavorable to BBs — this class of drugs continues to be widely used in clinical practice for the treatment of HTN. In a cross-sectional study published in 2024 by Prejbisz et al.,^38^ involving physicians from Italy, Poland, and Türkiye, approximately 23% of professionals reported prescribing BBs for HTN treatment, a proportion that increased to 30% when there were concomitant cardiovascular comorbidities.

This systematic review was developed based on several methodological premises. First, the antihypertensive effect of different substances within the BB class does not appear to be homogeneous, as some act only on β_1_ receptors, others on both β_1_ and β_2_ receptors, and some also exhibit vasodilatory effects.^6^ Therefore, grouping them into a single category while assuming the same antihypertensive effect may not be ideal. Second, combining multiple classes of antihypertensive drugs into a single comparator group significantly increases heterogeneity among studies, which meaningfully reduces confidence in the estimated effect. Third, conclusions should always be based on the comparative effect estimate (risk ratio), considering the previously defined MID, to determine whether a strategy produces a clinically relevant effect. Finally, it is essential to associate the estimated effect with the certainty of evidence assigned to each study supporting that result, preferably using the GRADE tool — which indicates how confident we can be in that information. If the quality is rated as low or very low, the actual effect in clinical practice may differ substantially from the observed estimate, underscoring the need for further RCTs to support any reliable conclusions.^39^

In light of these premises and with the aim of addressing the existing knowledge gap, the decision was made to compare atenolol individually — considered the most commonly prescribed BB for the treatment of HTN in Brazil — with other antihypertensive drug classes, analyzed separately. Surprisingly, only a limited number of studies were found evaluating this direct comparison, and none of the comparator drugs appeared in more than one original study. Overall, these studies were completed over 20 years ago, displayed considerable variability in methodological quality, and yielded imprecise results.

The network meta-analysis methodology employed in this review, although it did not include indirect or network comparisons in this case, offers the advantage of estimating the effect of atenolol with greater precision, without pooling various distinct classes of antihypertensive drugs, as commonly occurs in traditional meta-analyses. Moreover, no ranking of the different antihypertensive classes — a feature available in network meta-analysis — was generated, due to the high variability in the certainty of evidence across the original studies.

Among the comparisons that showed a favorable effect for other antihypertensive classes in the composite outcome of “cardiovascular events,” the most pronounced effect (hydrochlorothiazide combined with amiloride) was associated with very low certainty of evidence — suggesting that the true effect may differ substantially from the observed result. For the other two drug classes that demonstrated benefit (amlodipine and losartan), the confidence intervals touched the previously defined MID threshold, with the certainty of evidence rated as low and moderate, respectively.

Notably, some medical society recommendations against the use of atenolol are based on factors such as potency, dosage, frequency of administration, and pharmacokinetic interactions. In addition, daily doses of 100 to 200 mg have shown greater efficacy than 25 to 50 mg in patients with angina, which may also be relevant to the treatment of HTN.^40,41^

One fact that appears unequivocal is the magnitude of BP reduction in treatment arms using atenolol as the initial medication, compared to combinations administered in the comparator arms. In all included original studies, BP reduction was very similar between atenolol and the comparator drugs, suggesting a comparable antihypertensive effect across different drug classes.

This document presents several limitations. The most important is the small number of studies with direct comparisons between atenolol and other antihypertensive drug classes. To adequately answer the PICO question, more studies — ideally more recent and with high methodological quality — would be necessary. This limitation is even more pronounced in studies evaluating HTN treatment with other BBs, such as metoprolol, bisoprolol, carvedilol, and nebivolol, for which the scarcity of published articles precludes a reliable analysis. Second, although a network meta-analysis was conducted, the lack of indirect comparisons in this topic prevented the construction of an actual network of comparisons. Third, atenolol was not used as monotherapy in the included studies (Table 2). This reflects common clinical practice in the treatment of HTN, where drug combinations are frequently required to achieve target BP levels. However, such a characteristic limits the interpretation of the observed antihypertensive effect, making it difficult to determine how much of the benefit is attributable to atenolol alone versus co-administered medications. The same reasoning applies to comparator drugs, which were also not used as monotherapy in these studies. Because of the limited efficacy of monotherapy in achieving adequate BP control for most patients, it is unlikely that RCTs isolating the effect of a single medication with precision will be feasible.

**Table 2:** – Characteristics of the clinical trials included in this systematic review (highlighting the coadministered antihypertensive medications in each study)

Study	Year	Intervention	Additional antihypertensives	Primary outcomes	Secondary outcomes
ASCOT-BPLA	2005	Amlodipine	In the amlodipine group: perindopril; in the atenolol group: bendrofluazide and potassium	Fatal and nonfatal MI	Death, stroke, coronary events, CV events, cardiovascular procedures, heart failure
INVEST	2003	Verapamil	Trandolapril and/or hydrochlorothiazide	Death, nonfatal MI, and nonfatal stroke	Cardiovascular death, angina, adverse events, hospitalization, and BP control at 24 months
LIFE	2002	Losartan	Hydrochlorothiazide and other classes of antihypertensives	CV event	Death, hospitalization for heart failure
ELSA	2002	Lacidipine	Hydrochlorothiazide	IMT	Increase or reduction in carotid plaque (IMT ≥ 1.3 mm), cardiovascular death, and nonfatal CV event
UKPDS39	1998	Captopril	Furosemide, nifedipine, methyldopa, prazosin	Death and diabetes-related death	Macro- and microvascular complications, death, albuminuria, retinopathy, AMI, CAD, PAD, amputation
MRCOA	1992	Hydrochlorothiazide + Amiloride	If necessary, the intervention from the other arm was used; also nifedipine	Death, stroke, coronary events	N/A
COOPE	1986	No treatment	Bendrofluazide and methyldopa	Death, stroke, AMI	Symptoms

CAD: coronary artery disease; CV: cardiovascular; DM2: type 2 diabetes mellitus; IMT: intima-media thickness; AMI: acute myocardial infarction; N/A: not applicable; PAD: peripheral arterial disease.

These facts pose an additional challenge to the development of a Clinical Recommendation addressing the proposed PICO question — a response that is greatly needed, as the use of atenolol in the treatment of HTN remains a current, widespread practice.^42^

The members of the panel for this SBC Clinical Recommendation acknowledge that much of prior literature on this topic was interpreted during a time when the assessment of the quality of primary studies was not routinely applied. This may have contributed to an overestimation of the differences between BBs and other classes of antihypertensive agents in key cardiovascular outcomes. By incorporating the certainty of evidence, the difference between atenolol and other antihypertensive classes may be small, and that the reductions in BP values are comparable.

## Conclusion

HTN is a highly prevalent condition among adults, and its prevention and treatment should be priorities for any health care system. The findings of this review highlight the frequent need for combining antihypertensive agents in therapeutic regimens to achieve targets that prevent target organ damage and adverse outcomes.

Although the evidence is limited, atenolol showed similar effectiveness in lowering BP, with only minor differences in cardiovascular outcomes favoring other classes of antihypertensive drugs. Therefore, the expert panel recommends that atenolol may be considered as one of the options for combination therapy in the treatment of primary HTN in adults. Other BBs were not evaluated in this systematic review.

As with any clinical recommendation, prescribing decisions should be individualized and consider comorbidities, clinical characteristics, and patient preferences, along with the clinical judgment of the prescribing health professional.

**Table 1:** – Characteristics of the clinical trials included in this systematic review

Study	Year	Intervention	Follow-up (years)	Population	Age (mean)	Patients	Baseline mean BP (mmHg)	Final mean BP in treatment group (mmHg)	Final mean BP in atenolol group (mmHg)
ASCOT-BPLA (†)	2005	Amlodipine	5.5	HTN and ≥3 CVRF	40 to 79 (63)	19,257	164/94	136/77	137/79
INVEST	2003	Verapamil	2.7	HTN and CAD, >50 years	≥50 (66)	22,576	150/87 (//)	131.3/77	131/76.8
LIFE (*)	2002	Losartan	4.8	HTN and LVH	55 to 80	9,193	174/97	143.8/80.4	144.9/80.6
ELSA (‡)	2002	Lacidipine	3.75	HTN	45 to 75 (56)	2,334	163/101	141.4/85.5	141.2/85.4
UKPDS39	1998	Captopril	8.4	HTN + T2DM	26 to 65 (56)	1,148	160/94	144/83	143/81
MRCOA	1992	HCTZ + Amiloride	5.8	HTN	65 to 74 (70)	4,396	183/91	150/77	150/77
COOPE (§)	1986	No treatment	4.4	HTN	60 to 79 (65)	884	196/99	180/89	178/88

(*) CV events = total mortality; CV mortality + fatal and non-fatal AMI; fatal and non-fatal stroke. (†) It is not clearly defined whether MI and stroke results include only non-fatal events or also fatal ones. (‡) CV events defined as MI, stroke, and CV death only (excluding total mortality). (§) CV events = total mortality, total stroke, and total coronary disease. (//) Weighted average BP among patients using and not using anti-HTN. anti-HTN: antihypertensives; BP: blood pressure; CAD: coronary artery disease; CV: cardiovascular; CVRF: cardiovascular risk factors; HCTZ: hydrochlorothiazide; HTN: hypertension; LVH: left ventricular hypertrophy; MI: myocardial infarction; T2DM: type 2 diabetes mellitus.
